# KAT2A Promotes Hepatitis B Virus Transcription and Replication Through Epigenetic Regulation of cccDNA Minichromosome

**DOI:** 10.3389/fmicb.2021.795388

**Published:** 2022-01-24

**Authors:** Yi-Ping Qin, Hai-Bo Yu, Si-Yu Yuan, Zhen Yang, Fang Ren, Qing Wang, Fan Li, Ji-Hua Ren, Sheng-Tao Cheng, Yu-Jiao Zhou, Xin He, Hong-Zhong Zhou, Yuan Zhang, Ming Tan, Min-Li Yang, Da-Peng Zhang, Xu Wen, Mei-Ling Dong, Hui Zhang, Jing Liu, Zhi-Hong Li, Yao Chen, Ai-Long Huang, Wei-Xian Chen, Juan Chen

**Affiliations:** ^1^The Key Laboratory of Molecular Biology of Infectious Diseases Designated by the Chinese Ministry of Education, Chongqing Medical University, Chongqing, China; ^2^Department of Endocrine and Breast Surgery, The First Affiliated Hospital of Chongqing Medical University, Chongqing, China; ^3^Department of Medical Examination Centre, The Second Affiliated Hospital of Chongqing Medical University, Chongqing, China; ^4^Department of Clinical Laboratory, The Second Affiliated Hospital of Chongqing Medical University, Chongqing, China

**Keywords:** lysine acetyltransferase 2A (KAT2A), hepatitis B virus (HBV), covalently closed circular DNA (cccDNA), histone modification, succinylation

## Abstract

Hepatitis B virus (HBV) infection remains a major health problem worldwide. Sufficient maintenance of the HBV covalently closed circular DNA (cccDNA), which serves as a template for HBV transcription, is responsible for the failure of antiviral therapies. While accumulating evidence suggests that cccDNA transcription is regulated by epigenetic machinery, particularly the acetylation and methylation of cccDNA-bound histone 3 (H3) and histone 4 (H4), the potential contributions of histone succinylation and related host factors remain obscured. Here, by screening a series of succinyltransferases and desuccinylases, we identified KAT2A as an important host factor of HBV transcription and replication. By using HBV-infected cells and mouse models with HBV infection, KAT2A was found to affect the transcriptional activity of cccDNA but did not affect cccDNA production. Mechanism studies showed that KAT2A is mainly located in the nucleus and could bind to cccDNA through interaction with HBV core protein (HBc). Moreover, we confirmed histone H3K79 succinylation (H3K79succ) as a histone modification on cccDNA minichromosome by using the cccDNA ChIP-Seq approach. Importantly, KAT2A silencing specifically reduced the level of cccDNA-bound succinylated H3K79. In conclusion, KAT2A promotes HBV transcription and replication through epigenetic machinery, and our findings may provide new insight into the treatment of HBV infection.

## Introduction

Hepatitis B virus (HBV) infection remains a major global public health issue despite the availability of an effective vaccine and antiviral drugs that prevent HBV infection and arrest disease progression. Recent global hepatitis report estimates indicate 257 million people living with chronic HBV infection (Schweitzer et al., 2015; Salerno et al., 2020, p. 2). Moreover, among individuals with chronic hepatitis B (CHB) who are untreated, 15–40% develop cirrhosis and hepatocellular carcinoma (HCC) (Tang et al., 2018). HBV is a small hepatotropic virus with a 3.2-kb relaxed-circular partially double-stranded DNA (rcDNA) genome (Caballero et al., 2018). Upon entry into a hepatocyte, rcDNA in the HBV virion is delivered into the nucleus and converted into covalently closed circular DNA (cccDNA), which then serves as a transcription template for all viral transcripts, including the 3.5-kb precore RNA (pcRNA) and pregenomic RNA (pgRNA), the 2.4-kb and 2.1-kb surface mRNAs, and a 0.7-kb X mRNA (Block et al., 2007). pgRNA is a template for viral reverse transcription and translation into viral polymerase (Pol) and HBV core protein (HBc) (Jones and Hu, 2013). HBV replication begins with packaging of the pgRNA and Pol protein into core protein particles (nucleocapsids), then transforming pgRNA within the nucleocapsid into HBV core DNA (including ssDNA, dsDNA, and rcDNA). Eventually, mature rcDNA-containing nucleocapsids can re-deliver rcDNA to the nucleus to replenish the cccDNA pool or be secreted via interaction with envelope proteins as progeny virions. Therefore, HBV cccDNA is the key intermediate in the viral life cycle, which is responsible for the persistence of the infection during the natural course of chronic infection and prolonged antiviral treatment (Hong et al., 2017). Targeting cccDNA represents the key approach to cure HBV infection.

HBV cccDNA accumulates in the nucleus of infected cells as a stable episome and organises into a minichromosomes with histone 3 (H3) and histone 4 (H4) proteins and non-histone proteins, such as HBc, HBV x protein (HBx) and host transcription factors (Bock et al., 1994, 2001; Belloni et al., 2009). Accumulating evidence suggests that epigenetic modifications on cccDNA minichromosomes, such as histone modifications, play vital roles in the regulation of cccDNA transcriptional activity (Hong et al., 2017). Previous studies have suggested that the acetylation (ac) status of H3 and H4 on the HBV minichromosome is associated with cccDNA transcriptional activity, and that histone acetyltransferases CBP/p300 and deacetylase HDAC1 are involved in the regulation of HBV transcription (Pollicino et al., 2006; Belloni et al., 2009). A recent study reported that protein arginine methyltransferase 5 (PRMT5) could restrict HBV replication through epigenetic repression of cccDNA transcription and interference with encapsidation of pgRNA (Zhang et al., 2017, p. 5). Moreover, SIRT3 is a host factor epigenetically restricts HBV cccDNA transcription by acting cooperatively with histone methyltransferase (Ren et al., 2018, p. 3). Lysine succinylation (Ksucc), defined as the transfer of a succinyl group to a lysine residue of a protein, is a newly identified protein posttranslational modification (PTM) (Sreedhar et al., 2020). It has been found that histone succinylation has important roles in nucleosome and chromatin dynamics (Jing et al., 2018, 2020). At present, there are few reports about the regulation of histone succinylation on cccDNA minichromosomes (Yuan et al., 2020, p. 5; Yu et al., 2021, p. 7).

Here, by screening a series of succinyltransferases and desuccinylases, we demonstrate KAT2A, as an important host factor for HBV replication, catalyses the succinylation of H3K79 (H3K79succ) on cccDNA through its succinyltransferase activity to promote cccDNA transcription.

## Materials and Methods

### Cell Culture

HepG2 cells were obtained from American Type Culture Collection (ATCC) and the stabled cell line HepG2-NTCP (Na^+^/taurocholate co-transporting polypeptide) was constructed in our lab reference to previous study (Sun et al., 2017); Huh-7 cells were obtained from Health Science Research Resource Bank (HSRRB). HepAD38 cells were kindly provided by Prof. Ningshao Xia (The Xiamen University, Fujian, China). Primary human hepatocytes (PHHs) were acquired from ScienCell Research Laboratories; HepG2 and Huh-7 cells were maintained in Dulbecco’s modified Eagle medium (DMEM) (D6429, Sigma-Aldrich, United States) with 10% fetal bovine serum (FBS) (10270, Corning, New York, United States), 100 U/mL penicillin, and 100 μg/mL streptomycin (SV30010, HyClone, United States). HepG2-NTCP and HepAD38 cells were cultured in DMEM with 10% FBS and 400 μg/mL of G418 (345810, Merck Millipore, Germany). PHHs were maintained in hepatocyte medium (5201, ScienCell, United States). All cells were maintained in a humidified incubator at 37°C with 5% CO_2_.

### Plasmids, Antibodies, and Drugs

pCMV6-XL5-KAT2A was purchased from OriGene (SC125929). Lentivirus expressing KAT2A was purchased from Shanghai Genechem Company Limited (lentivector CMV-MCS-EF1-ZsGreen1-T2A-puromycin). Short hairpin RNA targeting KAT2A (shKAT2A-1 and shKAT2A-2) or non-targeting shRNA (shCont) were purchased from Shanghai Genechem Company Limited (lentivector hU6-MCS-ubiquitin-EGFP-IRES-puromycin). pCH9/3091 was presented by prof. Lin Lan (The Third Military Medical University, China). pCH9/3091 containing a 1.1-unit length HBV genome-wide (1818-3182(0)-1988 of HBV) (Genotype D, GenBank accession No. V01460) driven by the CMV promoter for HBV pgRNA transcription. Flag-HBc was constructed by in-frame insertion of full-length HBc into pcDNA3.1 that contains a Flag tag at the C-terminus. pcDNA3.1-HBc was constructed by in-frame insertion of full-length HBc into pcDNA3.1. pCH9/3091-ΔHBc, with a stop codon mutation in the HBc gene (codon 69) based on pCH9/3091. 3 × Flag-HBx was constructed by in-frame insertion of full-length HBx into p3 × Flag-CMV-7.1. The monomeric linearised HBV DNA was amplified from pCH9/3091 and purified by gel extraction kit (D2111, Magen, China). The primers used are listed in Supplementary Table 1. The siRNA and plasmid were transfected into cells by using Lipofectamine™ 3000 Transfection Reagent (L3000015, Thermo Fisher Scientific, United States). prcccDNA (the precursor plasmid of recombinant covalently closed circular DNA of HBV) and pCMV-Cre (encoding Cre recombinase) were obtained from Prof. Qiang Deng (Fudan University, Shanghai, China).

Mouse anti-p300 monoclonal antibody (NB100-616) and rabbit anti-HBsAg polyclonal antibody (NB100-62652) were obtained from Novus (United States). Rabbit anti-KAT2A monoclonal antibody (#3305S), rabbit anti-H3K14ac monoclonal antibody (#7627S), rabbit anti-CPT1A monoclonal antibody (#97361), rabbit anti-Flag monoclonal antibody (#14793), rabbit anti-SIRT5 monoclonal antibody (#8779), mouse anti-Histone H3 monoclonal antibody (#3638) and rabbit anti-GAPDH monoclonal antibody (#2118) were obtained from Cell Signaling Technology (United States). Rabbit anti-SIRT7 polyclonal antibody (S5947) was from Sigma-Aldrich (United States). Rabbit anti-HBV core antigen (HBcAg) polyclonal antibody (B0586) was obtained from Dako (United States). Mouse anti-HBcAg monoclonal antibody (sc-23945), mouse anti-GAPDH monoclonal antibody (sc-47724) and rabbit anti-β-actin monoclonal antibody (sc-47778) were obtained from Santa Cruz Biotechnology (United States). Rabbit anti-H3ac polyclonal antibody (06-599), rabbit anti-H3K9ac polyclonal antibody (06-942), normal rabbit IgG (NI01) and normal mouse IgG (12–371) were obtained from Merck Millipore (Germany). Rabbit anti-succinyllysine polyclonal antibody (PTM-401), rabbit anti-H3K122succ polyclonal antibody (PTM-413) and rabbit anti-H3K79succ polyclonal antibody (PTM-412) were obtained from PTM-Bio (China).

KAT2A inhibitor γ-butyrolactone (MB-3) (HY-129039) was obtained from MedChemExpress (United States).

### Virus Particles Production and Cell Infection

HBV particles were collected from the culture supernatant of HepAD38 cells. HBV wild type virus (HBV WT virus) were produced from Huh-7 cells transiently transfected with HBV expressing plasmid pCH9/3091 (containing a 1.1-unit length HBV genome driven by the CMV promoter). HBV HBc protein-deficient virus (HBV-ΔHBc virus) were collected from the supernatant of Huh-7 cells co-transfected with pCH9/3091-ΔHBc and vector expressing of HBc. Briefly, supernatants of cells were collected and mixed with polyethylene glycol (PEG)8000 at final concentration of 5%. Following gently rotated overnight at 4°C, the mixture was concentrated at 4,000 rpm for 30 min at 4°C to collect HBV particles. Centrifugal sediment was re-dissolved in serum-free opti-MEM at 1% volume of the original supernatant samples. For infection, cells were inoculated in plates. Then, the cells were infected with 1,000 vge/cell HBV particles diluted in culture medium supplemented with 2% dimethyl sulphoxide (DMSO) and 4% PEG8000. After 24 h post-infection, the cells were washed by phosphate buffer saline (PBS) three times.

### Real-Time Reverse-Transcription PCR

Total cellular RNA was extracted using TRNzol Universal reagent (DP424, TIANGEN, China) followed by DNase treatment. cDNA was synthesised from 1 μg extracted total RNA using an IScript™ cDNA Synthesis kit (KR116-02, TIANGEN, China). Relative quantification of target genes was determined by Fast Start Universal SYBR Green Master with β-actin mRNA as an internal control. The expression of target genes was calculated by the 2^–ΔΔ*Ct*^ method. The primers used are listed in Supplementary Table 1.

### Hirt Extraction of Hepatitis B Virus Covalently Closed Circular DNA and Analysis

Briefly, cells were lysed in 500 μL SDS lysis buffer (50 mM Tris-HCl, pH8.0, 10 mM EDTA, 150 mM NaCl, 1% SDS) at 37°C for 20 min. Then the cell lysate was mixed with 125 μL of 2.5 M KCl and rested overnight at 4°C. After centrifugation at 12,000 g for 20 min, the supernatant was collected (containing the cccDNA). Following purified by phenol/chloroform (1:1), the DNA was precipitated with ethanol and finally dissolved in TE buffer (10 mM Tris-HCl, pH7.5, 1 mM EDTA).

For liver samples, 10–20 mg liver tissues were lysed in lysis buffer (20 mM Tris-HCl, pH8.0, 5 mM EDTA, 400 mM NaCl, 1% SDS) by tissue homogenising apparatus. The cell lysate was incubated with 100 μg/mL protease K at 37°C overnight. Following purified by phenol/chloroform (1:1), DNA was precipitated with ethanol and finally dissolved in TE buffer.

Extracted DNA was digested by exonuclease V (M0345S, New England Biolabs, United States) for 30 min at 37°C. Taq-man probe PCR was carried out to quantify the cccDNA level with specific primers. The primers and probe used are listed in Supplementary Table 1.

### Hepatitis B Virus Core DNA Extraction and Quantification

Cells were lysed in 500 μL lysis buffer (10 mM Tris-HCl pH8.0, 1 mM EDTA, 1% NP-40, 2% sucrose) and incubated for 15 min at 37°C. The nuclei were removed by centrifugation at 16,000 g for 5 min. 10 μL of cytoplasmic cell lysate was put aside and β-actin was detected by western blot. The free nuclei acids in remaining cell lysates were digested with 40 IU/mL DNase I and 10 mM MgCl_2_ for 4 h at 37°C. After precipitation with 5% PEG8000, the HBV core capsids were incubated with proteinase K overnight at 45°C to release HBV DNA. Following purified by phenol/chloroform (1:1), the HBV DNA was precipitated with ethanol and finally dissolved in TE buffer.

For liver samples, 10–20 mg liver tissues were homogenised in 500 μL lysis buffer (10 mM Tris-HCl pH 8.0, 1 mM EDTA, 1% NP-40, 2% sucrose) by tissue homogenising apparatus and incubated for 15 min at 37°C. The subsequent steps are same as the above described. Extracted HBV DNA was subjected to absolute quantification PCR by using Fast Start Universal SYBR Green Master Mix. The primers used are listed in Supplementary Table 1.

### Southern Blotting

The DIG-High Prime DNA Labelling and Detection Starter Kit (11585614910, Roche, Germany) was used in the experiments. The HBV core DNA and Hirt-extracted DNA samples were subjected to 1% agarose gel electrophoresis and transferred onto a nylon membrane. The nylon membrane was cross-linked by exposure to UV light in a Stratalinker UV crosslinker and hybridised with a digoxigenin-labelled HBV full-length genomic DNA probe overnight at 42°C. The membrane was incubated in blocking solution for 30 min and antibody solution for another 30 min at 37°C. The signal was detected by exposing on an X-ray film. The mitochondrial gene Cox1 was hybridised as the loading control for HBV cccDNA.

For HBV DNA probe, full-length HBV DNA was amplified by PCR using pCH9/3091 plasmid as template. Electrophoresis of the PCR products was identified, and finally PCR product was purified by gel extraction kit (D2111-02, Magen, China) and PCR product purification kit (11732676001, Roche, Germany). Full-length HBV DNA was labelled with digoxigenin-11-dUTP with a kit supplied by Roche (11585614910). Labelling was carried out by following the manufacturers’ instructions.

### Northern Blotting

The DIG Northern Starter Kit (12039672910, Roche, Germany) was used in the experiments. Briefly, total cellular RNA was extracted and electrophoresed on 1.4% formaldehyde-agarose gel. The RNA was then transferred onto a nylon membrane and fixed by UV cross-linking. The nylon membrane was hybridised with a digoxigenin-labelled specific RNA probe (corresponding to nucleotides 126–1,225 of the HBV genome) overnight at 68°C. The membrane was incubated in blocking solution for 30 min and antibody solution for 30 min at 37°C. The signal was detected by exposing on an X-ray film.

For HBV RNA probe, three 500 bp HBV DNA fragments (corresponding to nucleotides 126–1,225 of the HBV genome) were amplified by PCR using pCH9/3091 plasmid as template. Electrophoresis of the PCR products was identified, and finally PCR product was purified by gel extraction kit (D2111-02, Magen, China) and PCR product purification kit (11732676001, Roche, Germany). Three 500 bp HBV DNA fragments (corresponding to nucleotides 126–1,225 of the HBV genome) were labelled with digoxigenin-11-UTP with a kit supplied by Roche (12039672910). Labelling was carried out by following the manufacturers’ instructions.

### Chromatin Immunoprecipitation

The chromatin immunoprecipitation (ChIP) assays were performed using Magna ChIP™ A/G Chromatin Immunoprecipitation Kit (17-10086, Merck Millipore, Germany) and with minor modifications (Belloni et al., 2012). Cells were scraped with cold PBS (containing protease inhibitor) and centrifuged at 1,000 g at 4°C for 5 min to pellet the cells. The cell pellets were resuspended in 500 μL ChIP Cell Lysis Buffer and the lysate was centrifuged to pellet the nuclei. The nuclei were fixed in 1% formaldehyde at 4°C for 30 min and neutralised by glycine. Isolated cross-linked nuclei were washed and resuspended in 500 μL Nuclear Lysis Buffer. Nuclear extract was sheared to ∼200–1,000 base pairs in length by sonication. After centrifugation, 50 μL of the supernatant was diluted 1:10 in dilution buffer and 5 μL of the sample was put aside as input, and the rest was then subjected to immunoprecipitation using the indicated antibodies and 20 μL protein A/G magnetic beads (17-10086, Merck Millipore, Germany) at 4°C with rotation for 14–16 h. Immunoprecipitation with normal IgG antibody was conducted in the experiment to exclude non-specific binding. The next day, bead-antibody/chromatin complex was washed by Low Salt Wash Buffer, High Salt Wash Buffer, LiCl Wash Buffer, TE Buffer, successively, and eluted in ChIP Elution Buffer containing Proteinase K at 62°C for 2 h with shaking. The immunoprecipitated chromatin was purified using Spin Columns according to the product manual (17-10086, Merck Millipore, Germany), and Taq-man probe qPCR with a specific cccDNA primer and probe was used to measure the level of cccDNA. Host genes glyceraldehyde-3-phosphate dehydrogenase (*GAPDH*) and cardiac gene myosin heavy chain 6 (*MYH6*) were used as control genes. The promoter regions of *GAPDH* and *MYH6* were targeted in the ChIP-PCR. The primers and probe used are listed in Supplementary Table 1.

For liver tissues, 25 mg liver tissues were finely minced by using the razor blade on ice and homogenised in tissue lysis buffer and the lysate was centrifuged to pellet the nuclei. The subsequent steps were the same as described above.

### Covalently Closed Circular DNA Chromatin Immunoprecipitation-Seq Assay

cccDNA ChIP-Seq assay was performed according to the method described previously with minor modifications (Tropberger et al., 2015). Briefly, HBV-infected PHHs were resuspended in cold nuclei isolation buffer (PBS with 0.1% Triton X-100, 0.1% NP-40, 1 mM DTT, 10 mM sodium butyrate, 1 × protease inhibitor) for 15 min on ice with gentle mixing every 5 min. After centrifugation at 1,000 g at 4°C for 5 min, the nuclei were pelleted and fixed in 1% formaldehyde for 30 min at 4°C. Cross-linked nuclei were washed and resuspended in Mnase digestion buffer (50 mM Tris-HCl pH7.5, 4 mM MgCl_2_, 1 mM CaCl_2_, 10% glycerol, 10 mM sodium butyrate, 1 × protease inhibitor). The nuclei were cut into mainly single nucleosome-sized pieces by digesting with 50 U/mL Mnase (New England Biolabs, United States) at 37°C for 10 min and ultrasonic crushing on ice. The sample was centrifugated at 6,500 g for 5 min and the supernatant was applied to 5–30% sucrose (sucrose, 50 mM Tris-HCl pH7.5, 50 mM NaCl, 5 mM EDTA pH8.0, 0.01% NP-40, 10 mM sodium butyrate, 1 × protease inhibitor) gradient centrifuge at 40,000 rpm at 4°C for 4 h. Mononucleosome containing fractions were pooled and subjected to ChIP assay. The immuno-enriched DNA sample was then used for sequencing.

### Co-immunoprecipitation and Western Blotting

The cells were lysed in IP lysis buffer (20 mM Tris (pH7.5), 150 mM NaCl, 1% Triton X-100, 20 mM sodium pyrophosphate, 20 mM β-glycerophosphate, 2 mM EDTA, 1 mM Na_3_VO_4_, 1 mM PMSF) containing 1 × protease inhibitors cocktail (04693159001, Roche, Germany) on ice for 20 min. After centrifugation, 500 μg of total protein was pre-cleared with protein A/G magnetic beads (88803, Thermo Fisher Scientific, United States) 1 h at 4°C. Then the protein complexes were immunoprecipitated with the indicated antibodies overnight at 4°C, followed by incubating with 20 μL protein A/G magnetic beads for another 2 h at room temperature. The protein complexes bound to the beads were washed three times with IP lysis buffer and eluted with loading buffer, followed by resolving on denaturing SDS/PAGE for Western blotting analysis. In general, the protein samples were separated by SDS-PAGE gel and transferred onto a PVDF membrane. Following being blocked in 5% non-fat milk, the membrane was incubated with primary antibody overnight at 4°C. The next day the membrane was incubated with a secondary antibody and the signal was visualised by ECL western blot reagents.

### Hepatitis B Virus Infection Mouse Model Involving Hepatitis B Virus Recombinant Covalently Closed Circular DNA Construction

All experiments using mice were carried out in the Laboratory Animal Centre of Chongqing Medical University. The animal study was reviewed and approved by Chongqing Medical University Institutional Animal Experimental Ethics Committee. Wild-type (wt) male mice (C57BL/6), aged 6–7 weeks, from the Laboratory Animal Centre of Chongqing Medical University were anaesthetised with ketamine and xylazine. For hydrodynamic injection, 4 μg prcccDNA and 4 μg pCMV-Cre plasmids were injected through tail veins (diluted to 8% of the mouse body weight with PBS) within 5–8 s (*n* = 20) (Yang et al., 2002, 2010; Huang et al., 2006). After 1 week, serum HBV DNA copies were determined by absolute quantification PCR. Next, mice were assigned to two groups randomly: negative group (1 × 10^8^ lentivirus-packaged control shRNA was injected into mice via the tail vein, *n* = 5). positive group (1 × 10^8^ lentivirus-packaged KAT2A shRNA was injected into mice via the tail vein, *n* = 5). Blood samples were collected via the orbital sinus every 2 days. At 8 days after treatment, the mice were euthanised by slow-fill CO_2_, and liver tissues were collected and immediately frozen in liquid nitrogen and stored at −80°C for further study.

### Immunofluorescence Staining

Cells grown on a coverslip were fixed in 4% paraformaldehyde for 15 min and then permeabilised in 0.5% Triton X-100 for 30 min. After blocking in PBS containing 2% bovine serum albumin (BSA) for 1 h, cells were incubated with the indicated antibodies overnight at 4°C. The cells were washed by PBS three times and incubated with secondary antibodies conjugated with Alexa Fluor 594 (goat anti-mouse, 1:1,000) and Alexa Fluor 488 (goat anti-rabbit, 1:400) for 2 h at room temperature. Then the cells were then incubated with 4′,6-diamidino-2-phenylindole (DAPI) for nuclear counterstaining, and images were captured by using a confocal laser scanning microscope (LEICA DMi8, Germany).

### Immunohistochemistry

Formalin-fixed and paraffin-embedded liver tissue from the mouse model were serially sectioned. Deparaffinisation was performed in xylene and dehydration in a gradient ethanol solution. Then, antigen retrieval was performed in a sodium citrate buffer (10 mmol/L, pH 6.0) using the microwaved heating method. The specimens were incubated with primary antibody overnight at 4°C. Then, the specimens were treated with a secondary antibody for 1 h at room temperature. Immunoreactivity was detected by using diaminobenzidine (DAB) staining. Counterstaining nuclei were performed using haematoxylin.

### Cell Counting Kit-8 Cell Viability Assay

The cells were mixed with CCK-8 (HY-K0301, MedChemExpress, United States) solutions and maintained for 2 h at 37°C. The amount of formazan generated by cellular mitochondrial dehydrogenase activity was measured at 450 nm by using Synergy H1 (BioTek Instruments, Inc.). The difference in cell viability was detected by comparing the absorbance of each well.

### Enzyme-Linked Immunosorbent Assay

The levels of HBsAg and HBeAg in the culture medium were assessed by using a direct double-antibody sandwich enzyme-linked immunosorbent assay (ELISA) according to the manufacturer’s protocol (KHB, Shanghai, China).

### Alanine Aminotransferase/Aspartate Aminotransferase Assay

The levels of alanine aminotransferase (ALT)/aspartate aminotransferase (AST) in liver tissue were detected by colorimetric microplate assay according to the manufacturer’s protocol (C009-2-1/C010-2-1, Nanjing Jiancheng Bioengineering Institute, China).

### Statistical Analysis

Statistics were performed with the non-parametric Mann-Whitney *U*-test. A value of *P* < 0.05 was considered significant. All statistical analyses were performed by using SPSS 19.0 software. Results are expressed as the average of three independent experiments. Data are shown as mean value ± standard error.

## Results

### Identification of Lysine Acetyltransferase 2A as a Host Factor Promoting Covalently Closed Circular DNA Transcription

**FIGURE 1:**
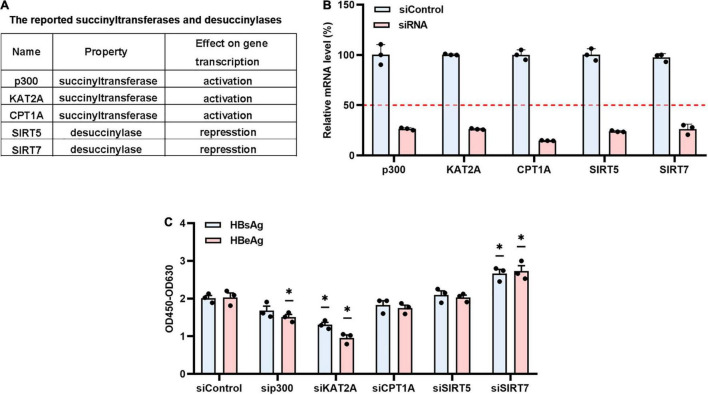
Identification of KAT2A as a host factor promoting cccDNA transcription. **(A)** The reported succinyltransferases and desuccinylases were summarised in the table. **(B)** After 4 days of siRNA treatment, total RNA was extracted by TRNzol Universal reagent. The silencing efficiency of related genes in Huh-7 cells was analysed by real-time PCR with specific primers. β-actin was used as the internal control. **(C)** The indicated siRNA (50 pmol) and monomeric linearised HBV DNA (1 μg) were co-transfected into huh-7 cells by using Lipofectamine™ 3000 Transfection Reagent. The supernatant was collected and HBeAg and HBsAg levels were detected by ELISA. *P < 0.05.

Multiple pieces of evidence have revealed the critical role of histone modification in regulating the transcription of cccDNA, particularly the acetylation and methylation of cccDNA-bound histone 3 (H3) and histone 4 (H4) (Zhang et al., 2017, p. 5; Ren et al., 2018, p. 3). To investigate whether histone succinylation plays a potential role in the HBV life cycle, we used the RNA interference method to screen a series of the reported histone succinyltransferases and desuccinylases, including p300, KAT2A, CPT1A, SIRT5, and SIRT7 (Figure 1A), which are known to catalyse histone succinylation or desuccinylation to result in transcription activation or repression (Zhang et al., 2011; Li et al., 2016; Wang et al., 2017; Kurmi et al., 2018; Ou et al., 2020). The data showed that the histone succinyltransferases and desuccinylases (including p300, KAT2A, CPT1A, SIRT5, and SIRT7) were efficiently knocked down (Figure 1B and Supplementary Figure 1A). Then the monomeric linear full-length HBV DNA genomes was transfected into Huh-7 cells (Supplementary Figure 1B), in which the cccDNA minichromosome could be formed and the level of the antigen in the supernatant was proven to serve as a surrogate marker for cccDNA transcription (Belloni et al., 2009). Our results suggested that KAT2A knockdown led to significant downregulation of both hepatitis B e antigen (HBeAg) and hepatitis B surface antigen (HBsAg) in the supernatant (Figure 1C). Therefore, we focused on KAT2A for further study.

### Lysine Acetyltransferase 2A Knockdown Suppresses Covalently Closed Circular DNA Transcription in an Hepatitis B Virus Infection Cell Model

**FIGURE 2:**
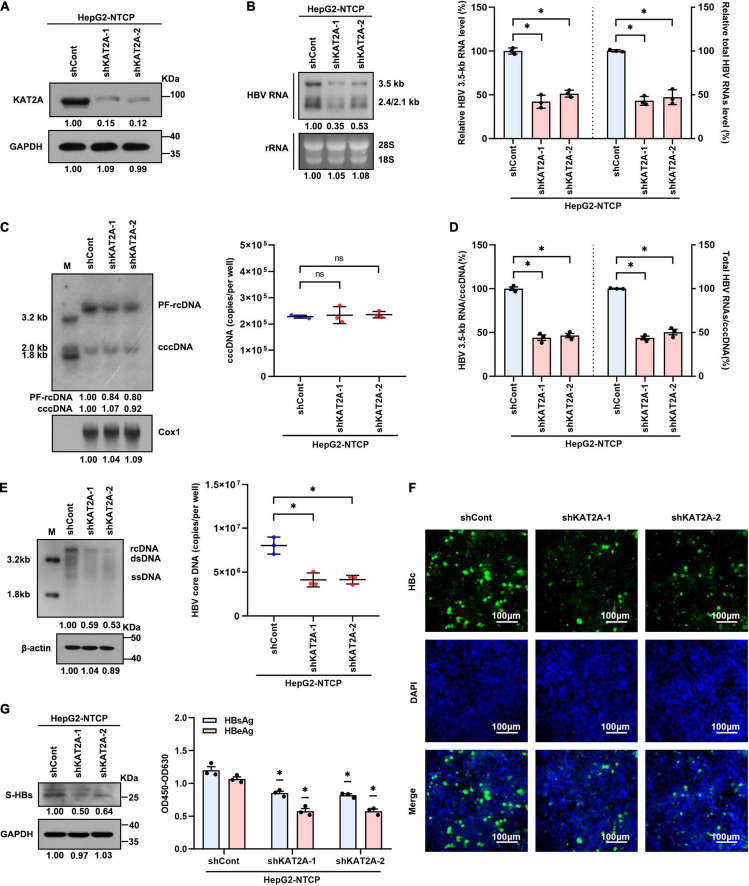
KAT2A knockdown suppresses cccDNA transcription in the HBV infection cell model. HepG2-NTCP cells were infected HBV particles at 1-day post shRNA transduction. At 4 days after infection. **(A)** The expression of KAT2A was detected by western blot. **(B)** Northern blotting and real-time PCR were used to analyse the effect of KAT2A knockdown on HBV RNAs. **(C)** HBV cccDNA was extracted by a modified Hirt DNA extraction protocol and then Southern blotting and Taq-man probe qPCR were used to detect the HBV cccDNA level. The mitochondrial gene Cox1 was hybridised as the loading control for HBV cccDNA. **(D)** The level of HBV 3.5-kb RNA, total HBV RNAs, and cccDNA were used to calculate the ratio of HBV 3.5-kb RNA to cccDNA and total HBV RNAs to cccDNA. **(E)** HBV core DNA was quantified by Southern blotting and absolute quantification PCR. The level of β-actin was used as a loading control for HBV core DNA. **(F)** HBV core protein (HBc) was produced by HBV infected HepG2-NTCP cells. Intracellular HBc was visualised by immunofluorescence staining. The scale bar is 100 μm. **(G)** Western blot was used to detect the level of HBsAg in cells (left). Cell culture supernatant was collected for HBsAg and HBeAg analysis via ELISA (right). Cox1, cyclooxygenase 1; rRNA, ribosomal RNA. *P < 0.05.

To confirm whether KAT2A is involved in HBV transcription and replication, the HepG2-NTCP cell model was used in our experiments. HepG2-NTCP cells were transduced with two individual lentiviruses expressing shRNA targeting KAT2A before HBV infection. Lentivirus-mediated shRNA effectively knocked down KAT2A and had no obvious cytotoxicity (Figure 2A and Supplementary Figure 2A). Knockdown of KAT2A distinctly reduced HBV 3.5-kb RNA and total HBV RNAs as evidenced by Northern blotting and real-time PCR (Figure 2B). Next, we investigated whether KAT2A could regulate the level of cccDNA, which is the only transcription template for all HBV RNAs in HepG2-NTCP cell model. Then, cccDNA was extracted and detected. Knockdown of KAT2A had no obvious effect on the level of cccDNA (Figure 2C and Supplementary Figure 2B), but could markedly silence cccDNA transcriptional activity, which was determined by the ratios of HBV 3.5-kb RNA to cccDNA or total HBV RNAs to cccDNA (Figure 2D). We also detected other HBV replicative intermediates including virus core DNA and proteins that are directly from cccDNA in HBV-infected HepG2-NTCP cells. Consistently, silencing KAT2A markedly reduced the level of HBV core DNA in the cytoplasm (Figure 2E), HBV core protein (HBc) in cytoplasm and nucleus (Figure 2F), surface antigen protein (S-HBs) in the intracellular space (Figure 2G, left), and HBeAg and HBsAg in the supernatant (Figure 2G, right). The results confirmed the role of KAT2A knockdown in the suppression of the cccDNA transcription and HBV replication.

### Lysine Acetyltransferase 2A Overexpression Promotes Hepatitis B Virus Transcription in Hepatitis B Virus Infection Cell Model

**FIGURE 3:**
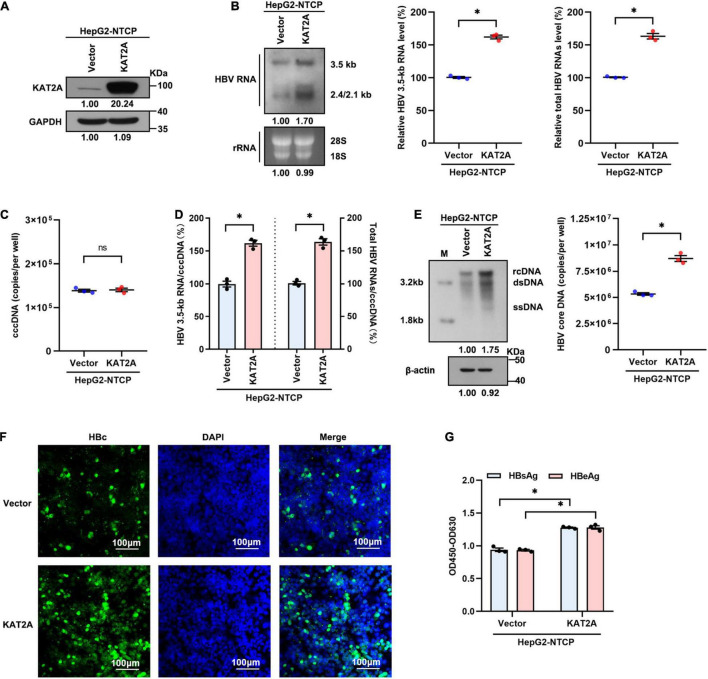
KAT2A overexpression promotes HBV transcription in the HBV infection cell model. HepG2-NTCP cells were infected HBV particles at 1-day post lentivirus expressing KAT2A plasmids transduction. At 4 days after infection. **(A)** The expression of KAT2A was detected by western blot. **(B)** The effect of KAT2A overexpression on HBV RNAs was analysed by Northern blotting and real-time PCR. **(C)** Taq-man probe qPCR was used to detect the HBV cccDNA level. **(D)** The level of HBV 3.5-kb RNA, total HBV RNAs, and cccDNA were used to calculate the ratio of HBV 3.5-kb RNA to cccDNA and total HBV RNAs to cccDNA. **(E)** HBV core DNA was quantified by Southern blotting and absolute quantification PCR. The level of β-actin was used as a loading control for HBV core DNA. **(F)** HBc was produced by HBV-infected HepG2-NTCP cells. Intracellular HBc was visualised by immunofluorescence staining. The scale bar is 100 μm. **(G)** Cell culture supernatant was collected for HBsAg and HBeAg analysis via ELISA. *P < 0.05.

We next transduced HepG2-NTCP cells with lentivirus expressing KAT2A before HBV infection to further determine the effect of KAT2A on HBV transcription and replication. Concordantly, overexpression of KAT2A significantly increased HBV RNA levels, as verified by Northern blotting and real-time PCR (Figures 3A,B). Overexpression of KAT2A also promoted cccDNA transcription without affecting the level of cccDNA (Figures 3C,D). Moreover, the levels of HBV core DNA in the cytoplasm (Figure 3E), HBc protein in the intracellular space (Figure 3F), and viral antigens in the supernatant (Figure 3G) were markedly increased after KAT2A overexpression. Together, these data suggest that KAT2A is a host factor that could promote HBV cccDNA transcription.

### Lysine Acetyltransferase 2A Bound to Covalently Closed Circular DNA and Regulated H3K79 Succinylation on the Covalently Closed Circular DNA Minichromosome

**FIGURE 4:**
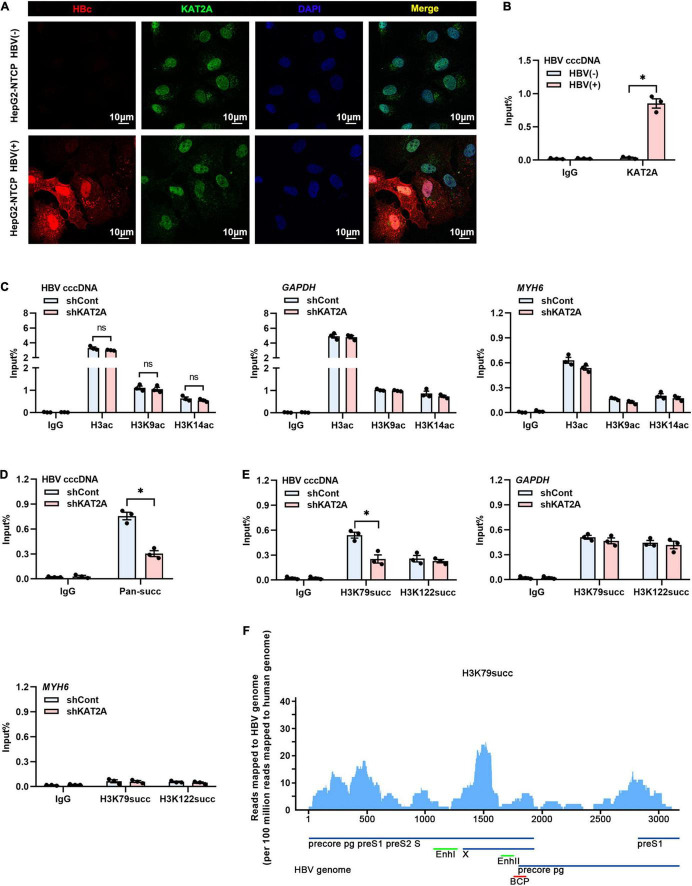
KAT2A bound to cccDNA and regulated H3K79 succinylation on the cccDNA minichromosome. **(A)** HepG2-NTCP cells were infected with HBV particles for 4 days, HBV core protein and endogenous KAT2A were observed by immunofluorescence assay using the specific antibodies. The scale bar is 10 μm. **(B)** Cross-linked chromatin from the HBV-infected and non-infected HepG2-NTCP nucleus was immunoprecipitated with a specific antibody or the control lgG. Taq-man probe qPCR was used to detect the HBV cccDNA level. ChIP results are expressed as% of input. **(C–E)** HepG2-NTCP cells were infected with HBV particles at 1-day post shKAT2A transduction. On 4 days post-infection. **(C)** The levels of H3ac, H3K9ac, and H3K14ac associated with cccDNA, GAPDH, or MYH6 promoter were analysed by ChIP assay with anti-H3ac, anti-H3K9ac, anti-H3K14ac, and the corresponding IgG, respectively. **(D)** ChIP assay was performed with anti-Pan-succ antibody. **(E)** The levels of H3K79succ and H3K122succ associated with cccDNA, GAPDH, or MYH6 promoter were analysed by ChIP assay with anti-H3K79succ, anti-H3K122succ, and the corresponding lgG, respectively. **(F)** ChIP-Seq analysis of the H3K79succ modification on the cccDNA minichromosome. The HBV-specific reads were quantified and normalised by the reads mapped to the human genome. *P < 0.05.

The obtained data encouraged us to investigate how KAT2A regulates cccDNA transcription. Considering that cccDNA transcription is a nuclear event, we first observed the subcellular localisation of KAT2A. Consistent with previous studies, KAT2A is a nuclear protein (Figure 4A). Following ChIP analysis, we found that KAT2A was recruited onto the cccDNA minichromosome after HBV infection (Figure 4B). As previously reported, KAT2A has acetyltransferase and succinyltransferase activity. KAT2A facilitates H3K9 and H3K14 acetylation, which are associated with transcription regulation (Benhamed et al., 2006; Hu et al., 2015; Fournier et al., 2016). It was also reported that H3K79succ could be catalysed by KAT2A and activate gene expression (Wang et al., 2017). Previous studies have shown that acetyl-CoA has a lower binding affinity to KAT2A than succinyl-CoA, and succinyl-CoA markedly reduces KAT2A-mediated acetylation (Wang et al., 2017). To further explore the role of the acetyltransferase function of KAT2A in cccDNA transcriptional regulation, HepG2-NTCP cells were treated with γ-butyrolactone (MB-3). MB-3 has been discovered as a small, cell-permeable KAT2A inhibitor with an IC_50_ of 100 μM, which can specifically reduce the acetylation of histone H3 catalysed by KAT2A (Mai et al., 2006). MB-3 at concentrations higher than 500 μM exhibited cell toxicity in HepG2-NTCP cells (CC50 > 800 μM) (Supplementary Figure 3A). H3K9 acetylation levels were reduced by MB-3 treatment and H3K79 succinylation levels were not affected at 12 and 24 h (Supplementary Figure 3B). We found that MB-3 treatment did not affect the level of HBV RNA (Supplementary Figure 3C). Then, a ChIP experiment was carried out after silencing KAT2A, and it was found that H3ac, H3K9ac, and H3K14ac modification on cccDNA histones was not affected compared with the control group (Figure 4C and Supplementary Figure 3D). Therefore, we preliminarily ruled out the role of the acetyltransferase function of KAT2A in cccDNA transcriptional regulation. Then, we asked whether the cccDNA minichromosome could undergo histone succinylation and be regulated by KAT2A. ChIP assays found that knockdown of KAT2A significantly reduced pan-succ (Figure 4D) and H3K79succ (Figure 4E) on the cccDNA minichromosome, and no effect was observed on H3K122succ (Figure 4E). The host genes GAPDH and MYH6 were chosen as the controls in the ChIP assay. Furthermore, we used HBV-infected primary human hepatocytes (PHHs) to prepare chromatin for the cccDNA ChIP-Seq assay as described previously (Tropberger et al., 2015). By mapping sequencing reads to the HBV genome, we confirmed H3K79succ as a histone modification on the cccDNA minichromosome (Figure 4F). Our results suggest that KAT2A binds to cccDNA and regulates H3K79 succinylation on the cccDNA minichromosome.

### Lysine Acetyltransferase 2A Binds to the Covalently Closed Circular DNA Minichromosome Through Interaction With HBc

**FIGURE 5:**
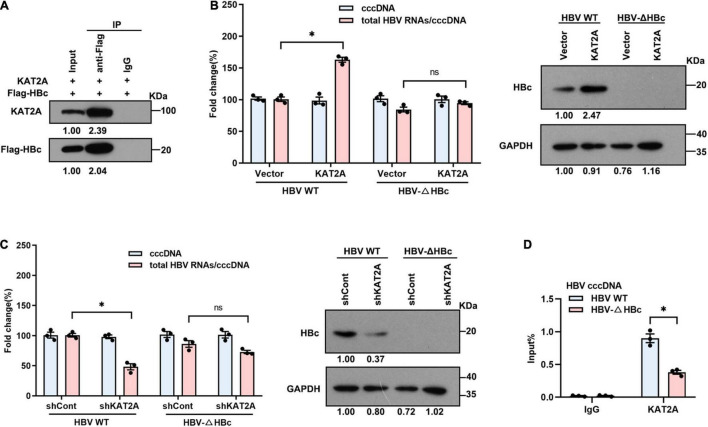
KAT2A binds to the cccDNA minichromosome through interaction with HBc. **(A)** HepG2-NTCP cells were co-transfected with plasmids encoding KAT2A and Flag-HBc for 3 days, the cells were subjected to Co-IP assay with the indicated antibody. The expression of the indicated proteins was analysed by western blot. **(B,C)** HepG2-NTCP cells were transduced with lentivirus expressing KAT2A or shKAT2A for 24 h, then infected with HBV wild type virus (HBV WT virus) and HBc-deficient virus (HBV-ΔHBc virus) for 4 days. Total HBV RNAs and cccDNA were extracted and quantified by real-time PCR and Taq-man probe qPCR for calculating the ratio of total HBV RNAs to cccDNA. The HBc proteins were detected by immunoblotting analysis. **(D)** HepG2-NTCP cells in 100 mm dishes were infected by HBV WT virus and HBV-ΔHBc virus. The cells were used for ChIP assays with the anti-KAT2A antibody. *P < 0.05.

Since the HBV HBc and HBV x protein (HBx) participate in the structural organisation of the cccDNA minichromosome (Bock et al., 2001; Belloni et al., 2009; Nassal, 2015), this may be the reason that KAT2A binds to the cccDNA minichromosome. Therefore, we further clarified whether there is an interaction between KAT2A and HBc or HBx protein. The Co-IP results showed that ectopically expressed KAT2A interacted with HBc (Figure 5A). However, no interaction between KAT2A and HBx was observed (Supplementary Figure 4A). In addition, HBc was mainly located in the nucleus and partially colocalised with KAT2A in HBV infected HepG2-NTCP cells (Figure 4A). To further explore the functional role of HBc in the binding of KAT2A to the cccDNA minichromosome, HBV wild type virus (HBV WT virus) and HBV HBc-deficient virus (HBV-ΔHBc virus) were collected from the supernatant of Huh-7 cells transfected with HBV wild type plasmids (HBV WT) or co-transfected with HBV-ΔHBc and a plasmid expressing HBc (Supplementary Figure 4B). After overexpression or silencing of KAT2A, the transcriptional activity of cccDNA could not be regulated in the group infected with HBV-ΔHBc virus (Figures 5B,C and Supplementary Figures 4C,D). Moreover, we found that the binding of KAT2A to the cccDNA minichromosomes was reduced in the HBV-ΔHBc group compared with the HBV WT group (Figure 5D). These results suggest that KAT2A may be targeted to HBV cccDNA minichromosome through its interaction with HBc.

### Antiviral Activity of Lysine Acetyltransferase 2A Knockdown *in vivo*

**FIGURE 6:**
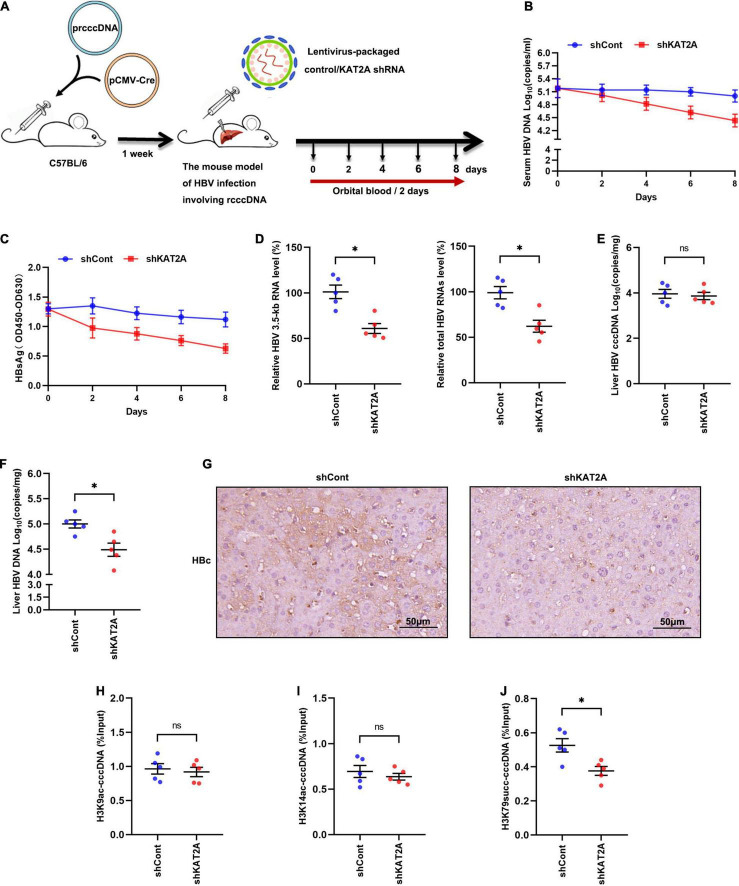
Antiviral activity of KAT2A knockdown *in vivo*. **(A)** Schematic depiction of experiments in C57BL/6 mice. **(B)** Serum HBV DNA was detected by absolute quantification PCR. **(C)** Serum HBsAg was measured via ELISA. **(D)** HBV 3.5-kb RNA and total HBV RNAs in liver tissue were measured by real-time PCR. **(E)** HBV cccDNA in liver tissue was detected by Taq-man probe qPCR. **(F)** The level of HBV DNA in liver tissue was detected by absolute quantification PCR. **(G)** HBc in liver tissues was analysed by IHC. The scale bar is 50 μm. **(H,I)** The levels of H3K9ac, H3K14ac associated with cccDNA were analysed by ChIP assay. **(J)** The level of H3K79succ associated with cccDNA was analysed by ChIP assay. *P < 0.05.

To investigate whether KAT2A silencing could inhibit HBV transcription *in vivo*. A mouse model of HBV infection involving the generation of HBV recombinant cccDNA (rcccDNA) using site-specific DNA recombination techniques was used (Qi et al., 2014; Supplementary Figure 5A). The workflow of the mouse model was shown (Figure 6A), wild-type (wt) male mice (C57BL/6) were hydrodynamically injected with prcccDNA and pCMV-Cre, which resulted in the accumulation of nuclear cccDNA that was epigenetically organised as a minichromosome. Then the wt C57BL/6 mice were hydrodynamically injected with 1 × 10^8^ lentivirus-packaged control/KAT2A shRNA for 8 days. Body weight was monitored every 2 days, all animals were sacrificed to collect liver tissue on day 8, liver weight was measured. There were no significant differences in body weight or liver weight in shKAT2A-treated mice compared to the control group (Supplementary Figures 5B,C). The expression of KAT2A, the level of ALT/AST were detected in liver tissues. The data showed that lentivirus-mediated shRNA could effectively knock down KAT2A and had no significant hepatotoxicity (Supplementary Figures 5D,E). Serum viral markers were detected during treatment, and our results showed that silencing KAT2A decreased the serum HBV DNA levels (Figure 6B), as well as HBsAg (Figure 6C). We further evaluate the effect of silencing KAT2A on intrahepatic HBV RNAs, cccDNA, HBV DNA, and HBc protein. It turned out that silencing KAT2A reduced HBV 3.5-kb RNA and total HBV RNAs (Figure 6D) without affecting the level of HBV cccDNA (Figure 6E). Consistently, silencing KAT2A reduced HBV DNA levels (Figure 6F). Immunohistochemical analysis showed that that the expression of HBc was inhibited by KAT2A silencing (Figure 6G). Interestingly, the cccDNA-ChIP assay was carried out in liver tissue and found that silencing KAT2A did not affect levels of H3K9ac and H3K14ac modification on the cccDNA minichromosome (Figures 6H,I), but decreased the level of H3K79succ (Figure 6J). Taken together, these results demonstrated that silencing KAT2A could restrict HBV transcription and replication *in vivo*, and our study may provide new insight into the treatment of HBV infection.

## Discussion

Sufficient maintenance of the HBV covalently closed circular DNA (cccDNA), which serves as a template for HBV transcription, is responsible for the failure of antiviral therapies. Identification of the molecular mechanisms regulating cccDNA transcriptional activity may reveal new potential therapeutic targets for anti-HBV drugs. Multiple pieces of evidence have revealed the critical role of histone modification in regulating the transcription of cccDNA (Zhang et al., 2017, p. 5; Ren et al., 2018, p. 3). For example, histone acetylation and methylation have been well characterised (Belloni et al., 2012; Palumbo et al., 2015; Rivière et al., 2015; Yuan et al., 2019). Succinylation is a recently discovered PTM. It has been reported that succinylation, acetylation, and monomethylation of lysine residues change the charge state from + 1 to −1, and from + 1 to 0, and do not change the charge state at all, respectively. Therefore, succinylation may lead to more substantial changes in the structure and function of proteins than acetylation and methylation (Wang et al., 2017). Moreover, succinylation is also a frequently occurring histone modification that is central to the regulation of chromatin-based processes (Zhang et al., 2011; Weinert et al., 2013). However, at present, there are few reports on the regulation of cccDNA minichromosomes by histone succinylation (Yuan et al., 2020, p. 5; Yu et al., 2021, p. 7). Our previous study had identified that SIRT7 inhibited HBV transcription and replication by catalysing cccDNA-bound histone H3K122 desuccinylation (Yu et al., 2021), indicating that histone succinylation may play a crucial role in cccDNA transcription.

Previous studies demonstrated that KAT2A histone succinyltransferase coupled with the α-ketoglutarate dehydrogenase (α-KGDH) complex regulated H3K79 succinylation and gene expression (Wang et al., 2017) and KAT2A could be recruited to the HBV cccDNA minichromosome, thus contributing to HBV replication (Belloni et al., 2009). Recently, a paper by Yuan et al. came to a similar conclusion: KAT2A-mediated succinylation of histone H3K79 contributed to the epigenetic regulation of cccDNA minichromosomes, thus was beneficial to HBV replication (Yuan et al., 2020, p. 5). They also concluded that IFN-α effectively depressed histone H3K79 succinylation in the HBV cccDNA minichromosome. However, the mechanism by which KAT2A is recruited to the HBV cccDNA minichromosome remains elusive. In our study, we proved that KAT2A could bind to cccDNA through interaction with HBc where it catalysed H3K79 succinylation. Moreover, we confirmed histone H3K79 succinylation as a histone modification on the cccDNA minichromosome by using the cccDNA ChIP-Seq approach. Since the effect of KAT2A on HBV replication has not been validated *in vivo*, our data demonstrated that KAT2A modulated histone H3K79 succinylation on cccDNA minichromosome to enhance HBV transcription and replication *in vivo*.

HBV cccDNA exists in the nucleus as the template for HBV RNA transcription. One previous study showed that the α-KGDH complex interacted with KAT2A in the nucleus (Wang et al., 2017). In our study, we validated the subcellular localisation of KAT2A in HepG2-NTCP cells. And similarly, we found that KAT2A was a nuclear protein which might be related to cccDNA minichromosomes. The viral proteins HBc and HBx have been shown to be associated with the cccDNA minichromosome and thus were candidates to mediate the preferential binding of KAT2A to cccDNA (Lucifora et al., 2014; Zhang et al., 2017, p. 5). Therefore, we further found that ectopically expressed KAT2A co-precipitated with HBc rather than HBx. More importantly, compared with HBV-WT virus infected cells, the recruitment of KAT2A to cccDNA was markedly reduced in HBV-ΔHBc virus infected cells (Figure 5D). Together, these results suggest that HBc plays a functional role in the regulation of KAT2A on cccDNA transcription.

In conclusion, the present study revealed that KAT2A could bind to cccDNA through interaction with HBc where it catalysed H3K79 succinylation, thus facilitated HBV transcription and replication. These findings broaden our knowledge regarding the physiological functions of KAT2A and the roles of histone succinylation in epigenetic regulation of HBV cccDNA. In addition, our results may reveal new potential therapeutic targets for anti-HBV drugs and hence assist in the design of strategies aimed at silencing and eventually depleting the cccDNA reservoir.

## Data Availability Statement

The datasets presented in this study can be found in online repositories. The names of the repository/repositories and accession number(s) can be found below: https://www.ncbi.nlm.nih.gov/geo/query/acc.cgi?acc=GSE188634.

## Ethics Statement

The animal study was reviewed and approved by the Chongqing Medical University Institutional Animal Experimental Ethics Committee, Chongqing Medical University, Chongqing, China.

## Author Contributions

JC designed the study. Y-PQ, H-BY, S-YY, and ZY performed the experiments. FL, J-HR, S-TC, and FR acquired the data. YZ, MT, Y-JZ, M-LD, HZ, XW, and A-LH analysed the data. Y-PQ and H-BY wrote the manuscript. All authors contributed to manuscript revision, read, and approved the submitted version.

## Conflict of Interest

The authors declare that the research was conducted in the absence of any commercial or financial relationships that could be construed as a potential conflict of interest.

## Publisher’s Note

All claims expressed in this article are solely those of the authors and do not necessarily represent those of their affiliated organizations, or those of the publisher, the editors and the reviewers. Any product that may be evaluated in this article, or claim that may be made by its manufacturer, is not guaranteed or endorsed by the publisher.

## Abbreviations

KAT2A: Lysine acetyltransferase 2A
HBV: Hepatitis B virus
cccDNA: covalently closed circular DNA
rcDNA: relaxed-circular partially double-stranded DNA
pcRNA: precore RNA
pgRNA: pregenomic RNA
Pol: viral polymerase
HBeAg: Hepatitis B e antigen
HBsAg: Hepatitis B surface antigen
HBc: HBV core protein
HBx: HBV x protein
nucleocapsids: core protein particles
PHH: Primary human hepatocytes
PTM: posttranslational modification
ac: acetylation
Ksucc: Lysine succinylation
H3K79succ: Succinylation of histone H3K79
ChIP: Chromatin immunoprecipitation
Co-IP: Co-immunoprecipitation
H3: Histone 3
H4: Histone 4
GAPDH: Glyceraldehyde-3-phosphate dehydrogenase
MYH6: Myosin heavy chain 6.
