# Supplementary material for: KAT2A Promotes Hepatitis B Virus Transcription and Replication Through Epigenetic Regulation of cccDNA Minichromosome
Source: Front Microbiol. 2022 Jan 24;12:795388. doi: 10.3389/fmicb.2021.795388 (PMC8818952; doi:10.3389/fmicb.2021.795388)
Supplement: Supplementary file 1 [file Data_Sheet_1.PDF]

KAT2A promotes hepatitis B virus transcription and replication through epigenetic regulation of cccDNA minichromosome

Supplementary materials and methods
Fig. S1
Fig. S2
Fig. S3
Fig. S4
Fig. S5
Table. S1
Original data 1
Original data 2
Original data 3
Original data 4
Original data 5
Original data 6

Fig. S1

Supplementary Figure 1. Validation of silent efficiency. (A) After 4 days of siRNA treatment, total protein was extracted. Western blot was used to detect the silencing efficiency of relevant genes in Huh-7 cells. GAPDH was used as the loading control. (B) The scheme of producing the monomeric linearized HBV DNA.

Fig. S2

Supplementary Figure 2. KAT2A knockdown suppresses cccDNA transcription in the HBV infection cell model. (A) 5×10^4 cells were plated in each well of a 48-well plate, the cytotoxicity of lentivirus expressing shRNA on HepG2-NTCP cells was investigated by CCK-8 assay and microscope on 5 days post shRNA transduction. (B) The EcoRI undigested-DNA and EcoRI digested-DNA were examined by Southern blot analysis to verify the cccDNA and PF-rcDNA in the cells, and the linearized HBV DNA at 3.2 kb position could be detected.

Fig. S3

Supplementary Figure 3. KAT2A acetyltransferase inhibitor MB-3 did not change levels of HBV 3.5-kb and total transcripts. (A) HepG2-NTCP cells were treated with MB-3 at a series concentration for 72 h. Effects of MB-3 on the cell viability were determined by the MTT assay. (B) HepG2-NTCP cells were treated for 12h or 24h with 100 µM MB-3 or DMSO as control, and the level of H3K9ac and H3K79succ were analysed by western blot using a specific antibody. Histone 3 (H3) was used as the loading control. (C) On 4 days post-infection, HepG2-NTCP cells incubated with KAT2A inhibitors MB-3 (100 µM) at the indicated times, the level of HBV 3.5-kb RNA, total HBV RNAs were detected by real-time PCR. (D) HepG2-NTCP cells were infected with HBV particles at 1-day post shKAT2A

transduction. On 4 days post-infection, the level of KAT2A was detected by western blot.

Fig. S4

Supplementary Figure 4. KAT2A binds to the cccDNA minichromosome through interaction with HBc. (A) HepG2-NTCP cells were co-transfected with plasmids encoding KAT2A and 3×Flag-HBx, the cells were subjected to Co-IP assay with the indicated antibody. The expression of the indicated proteins was analyzed by western blot. (B) Schema of HBV wild type virus (HBV-WT virus) and HBV HBc-deficient virus (HBV-ΔHBc virus) were collected from the supernatant of Huh-7 cells transfected with pCH9/3091 or co-transfected with pCH9/3091-ΔHBc and pcDNA3.1-HBc. (C-D) HepG2-NTCP cells were transduced with lentivirus expressing KAT2A or shKAT2A for 24h, then infected with HBV wild type virus (HBV WT virus) and HBc-deficient virus (HBV-ΔHBc virus) for 4 days. The KAT2A proteins were detected by immunoblotting analysis.

Fig. S5

Supplementary Figure 5. Antiviral activity of KAT2A knockdown in vivo. (A) Schema of Cre/loxP-mediated recombination of a precursor plasmid (i.e., prcccDNA) excising an rcccDNA episome with a loxP-chimeric intron. loxP-chimeric intron spliced from viral transcripts during RNA processing, without disturbing the replication cycle of HBV. (B) After treatment with lentivirus-packaged Control/KAT2A shRNA, body weight was monitored every two days. (C) Liver and body weight were monitored on day 15, and the ratio of liver to body weight was calculated. (D) The expression of KAT2A in liver tissue was detected by western blot. (E) The levels of alanine aminotransferase (ALT) and aspartate aminotransferase (AST) in liver tissue were determined by a colorimetric microplate assay.

Table S1:

Supplementary Table 1. Primers and siRNA Sequences

Gene	Primer Sequences (5’→3’)
HBV cccDNA-F	GTGCACTTCGCTTCACCTCT
HBV cccDNA-R	AGCTTGGAGGCTTGAACAGT
HBV cccDNA probe	ACGTCGCATGGAGACCACCGTGAACGCC
HBV 3.5-kb RNA-F	GCCTTAGAGTCTCCTGAGCA
HBV 3.5-kb RNA-R	GAGGGAGTTCTTCTTCTAGG
Total HBV RNA-F	ACCGACCTTGAGGCATACTT
Total HBV RNA-R	GCCTACAGCCTCCTAGTACA
β-actin-F	CTCTTCCAGCCTTCCTTCCT
β-actin-R	AGCACTGTGTTGGCGTACAG
β-actin mRNA (mouse)-F	CCACCATGTACCCAGGCATT
β-actin mRNA (mouse)-R	CGGACTCATCGTACTCCTGC
HBV DNA-F	CCTAGTAGTCAGTTATGTCAAC
HBV DNA-R	TCTATAAGCTGGAGGAGTGCGA
F1824-1845	TTTTTCACCTCTGCCTAATCATCTC
R1823-1804	GTTGCATGGTGCTGGTGCGCA
p300-F	AGCCAAGCGGCCTAAACTC
p300-R	TCACCACCATTGGTTAGTCCC
KAT2A-F	CAGTTTCGGCAGAGGTCTCA
KAT2A-R	ATGAGTGGTTTCGTAGCGGG
CPT1A-F	ATCAATCGGACTCTGGAAACGG
CPT1A-R	TCAGGGAGTAGCGCATGGT
SIRT5-F	CTGGATCCTGCCATTCTGGA
SIRT5-R	CTGGGTACACCACAGAGGAA
SIRT7-F	CAGGGAGTACGTGCGGGTGT
SIRT7-R	TCGGTCGCCGCTTCCCAGTT
GAPDH-ChIP-F	TACTAGCGGTTTTACGGGCG
GAPDH-ChIP-R	TCGAACAGGAGGAGCAGAGAGCGA
MYH6-ChIP-F	AGAAGCTGCGCTCAGACCTGTCTCG
MYH6-ChIP-R	TCCAGGTCCCGCCGCATCTT
siRNA	Target Sequences (5’→3’)
p300-siRNA	CCAAGTGGCACACTGTGCATCTTCT
KAT2A-siRNA	CAAUGAAACCUGUAAGUGUTT
CPT1A-siRNA	CAGCACATGAGAGACAGCAAGCACA
SIRT5-siRNA	CCTCTTGTGGAGTTGTGGCTGAGAA
SIRT7-siRNA	CGGAACGCCAAATACTTGGTCGTCT
