# Supplementary figures and images for: KAT2A Promotes Hepatitis B Virus Transcription and Replication Through Epigenetic Regulation of cccDNA Minichromosome
Source: Front Microbiol. 2022 Jan 24;12:795388. doi: 10.3389/fmicb.2021.795388 (PMC8818952; doi:10.3389/fmicb.2021.795388)

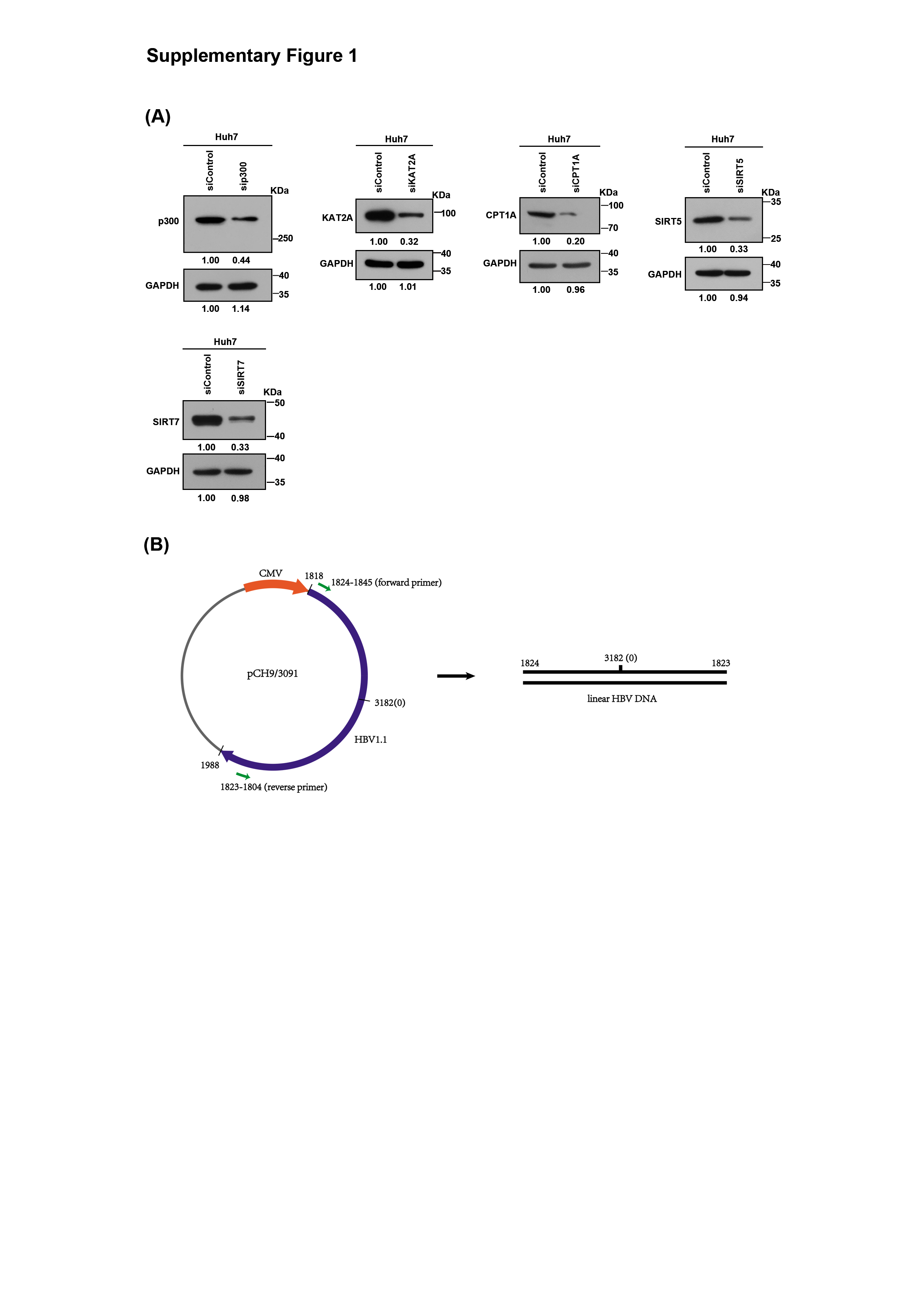

Supplement: Supplementary file 2 [file Image_1.TIF]

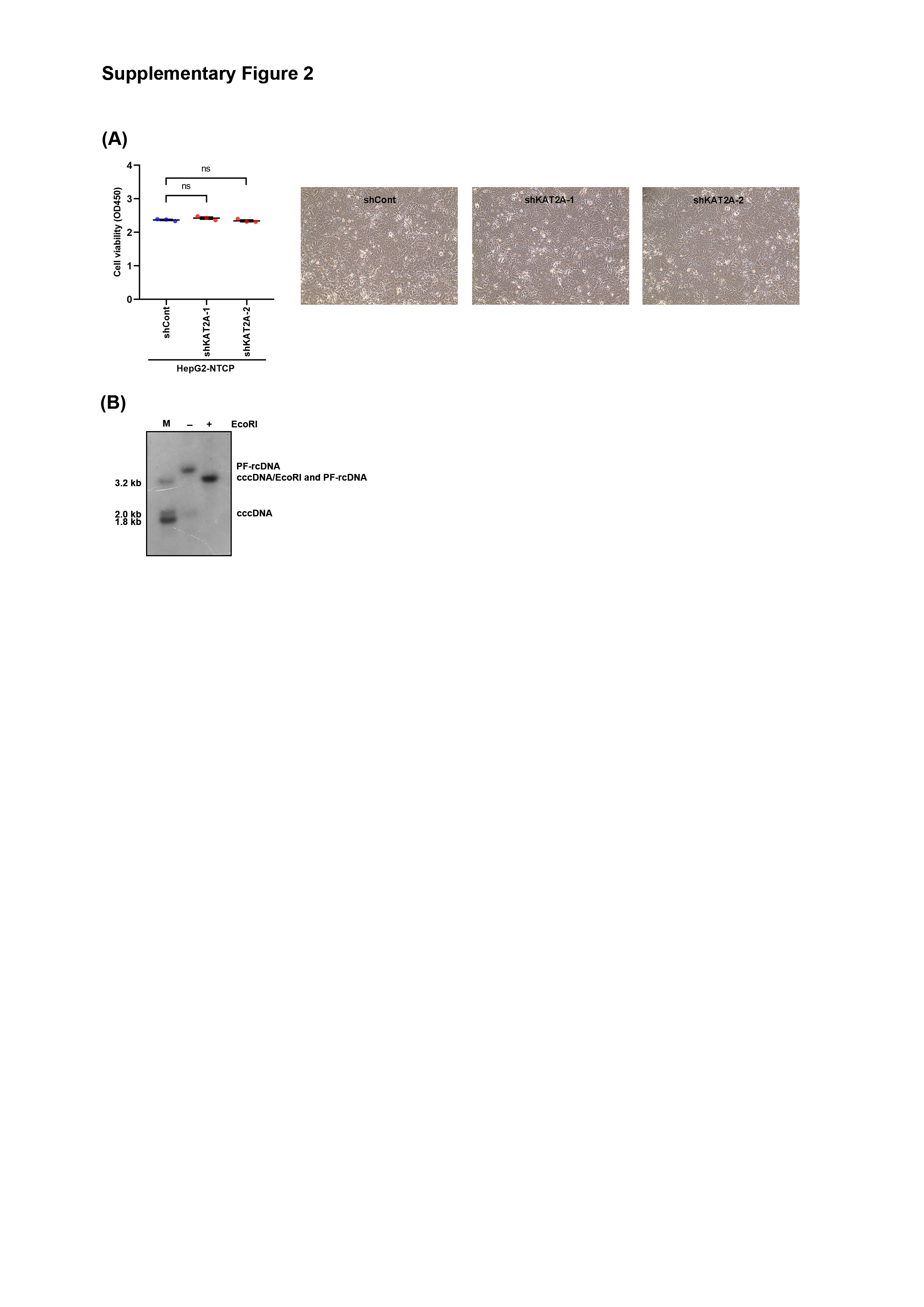

Supplement: Supplementary file 3 [file Image_2.TIF]

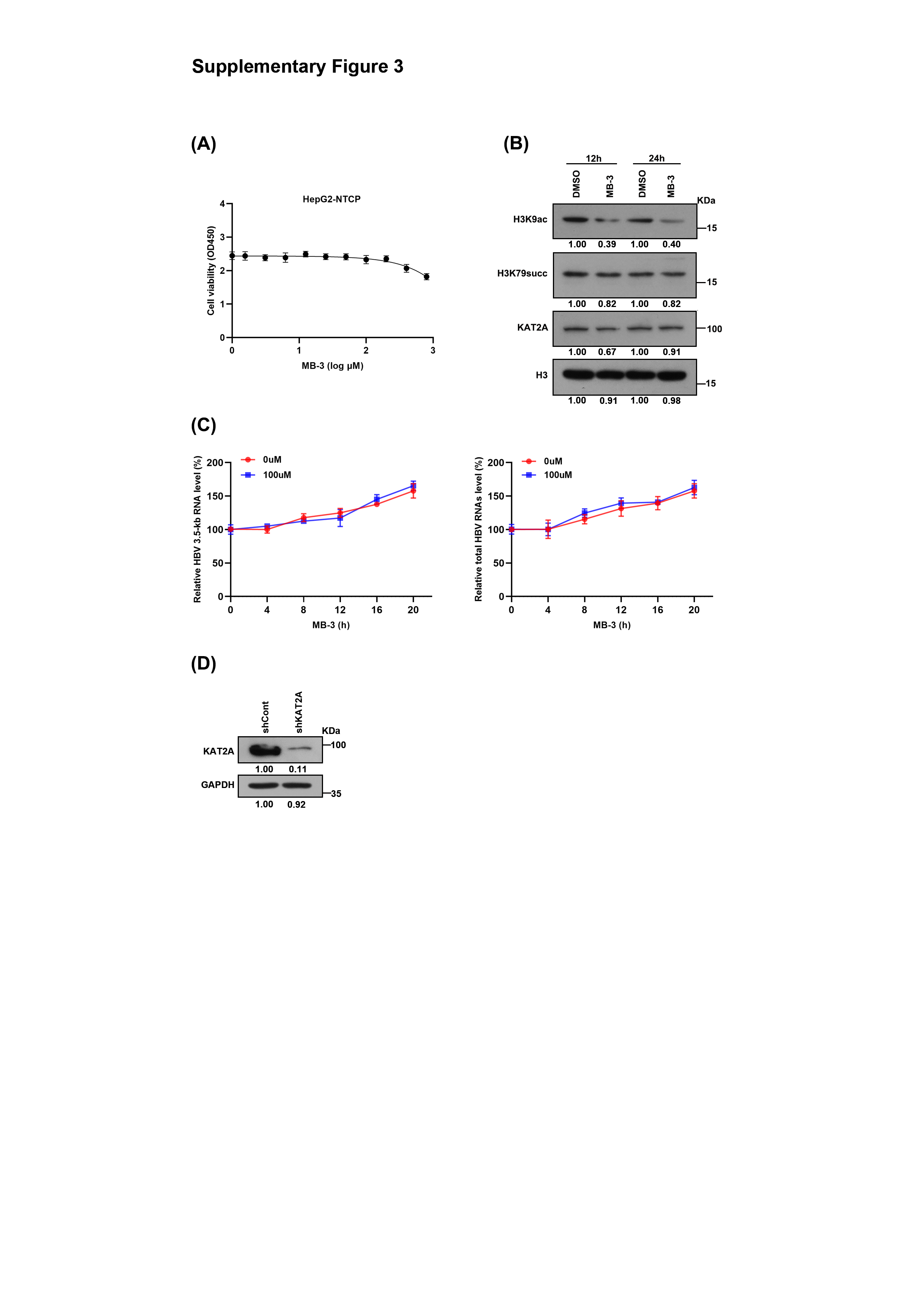

Supplement: Supplementary file 4 [file Image_3.TIF]

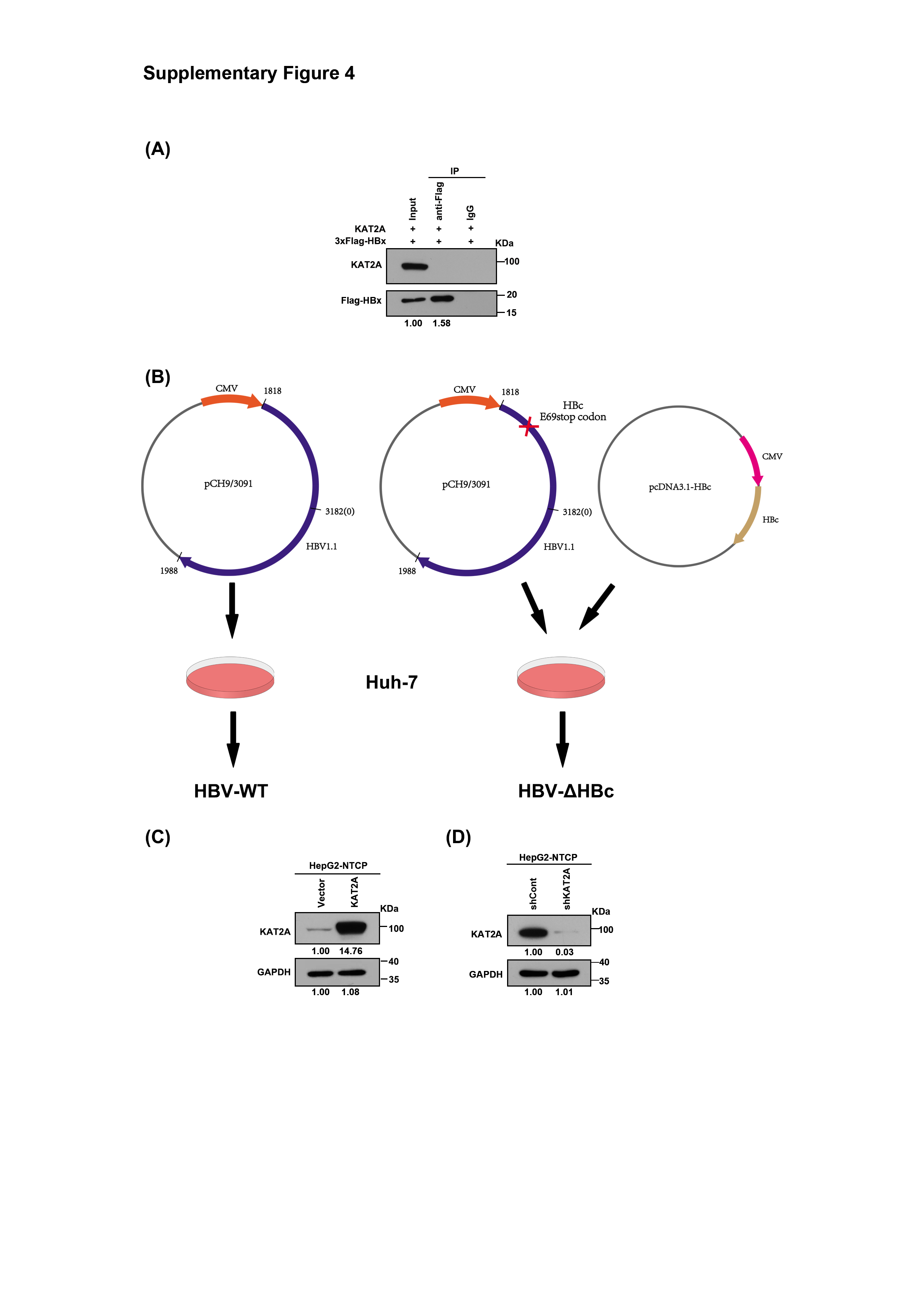

Supplement: Supplementary file 5 [file Image_4.TIF]

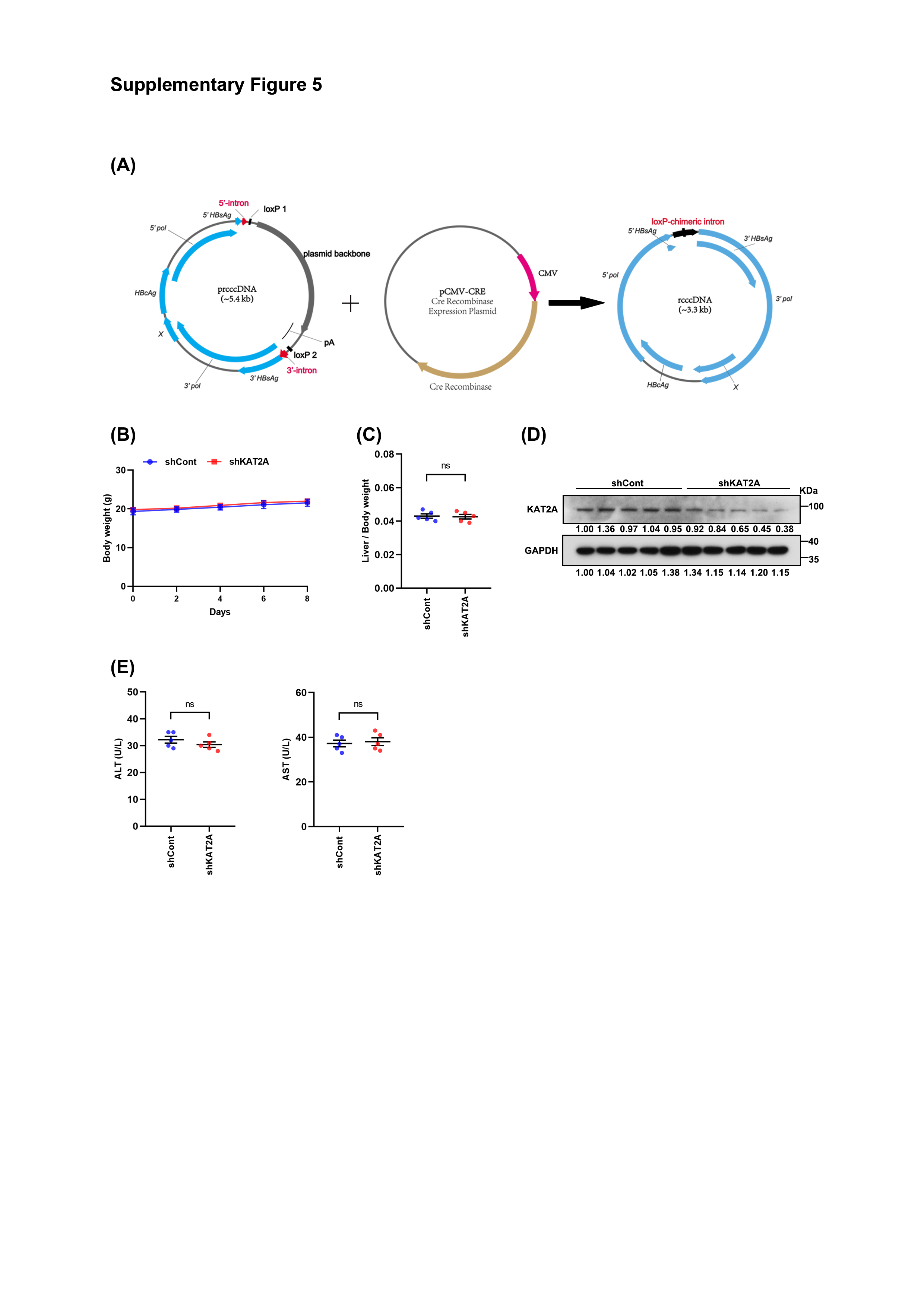

Supplement: Supplementary file 6 [file Image_5.TIF]

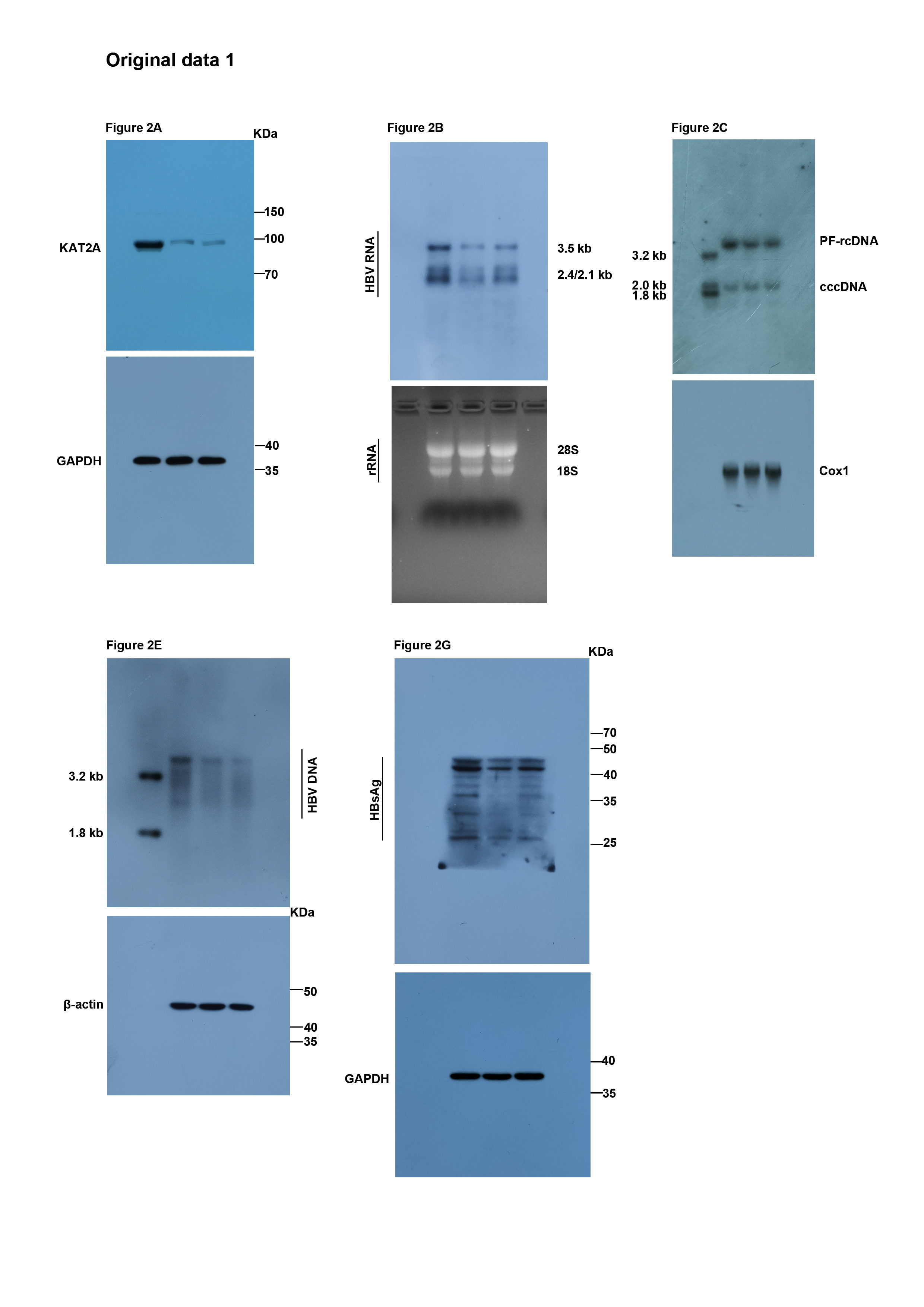

Supplement: Supplementary file 7 [file Image_6.TIF]

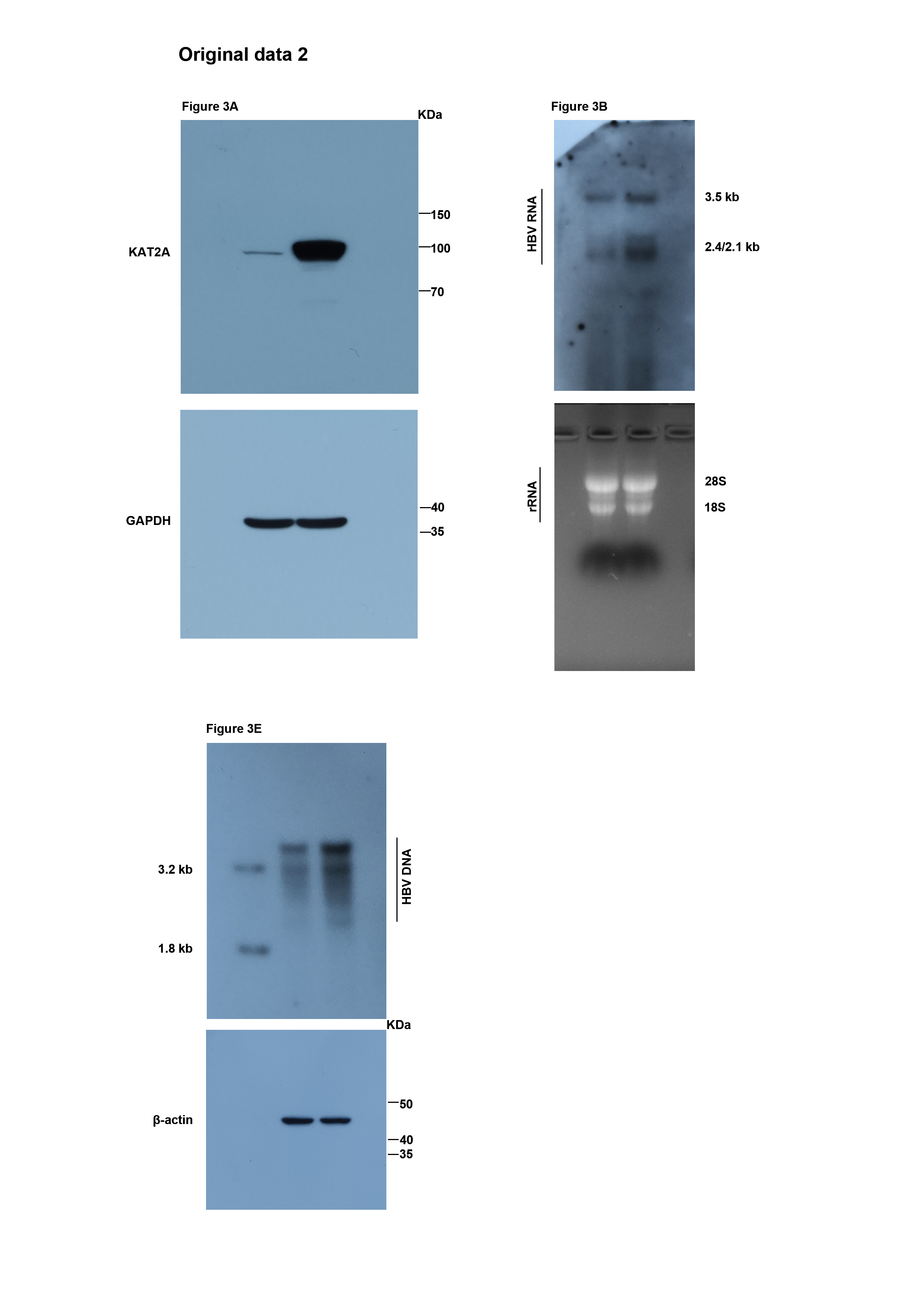

Supplement: Supplementary file 8 [file Image_7.TIF]

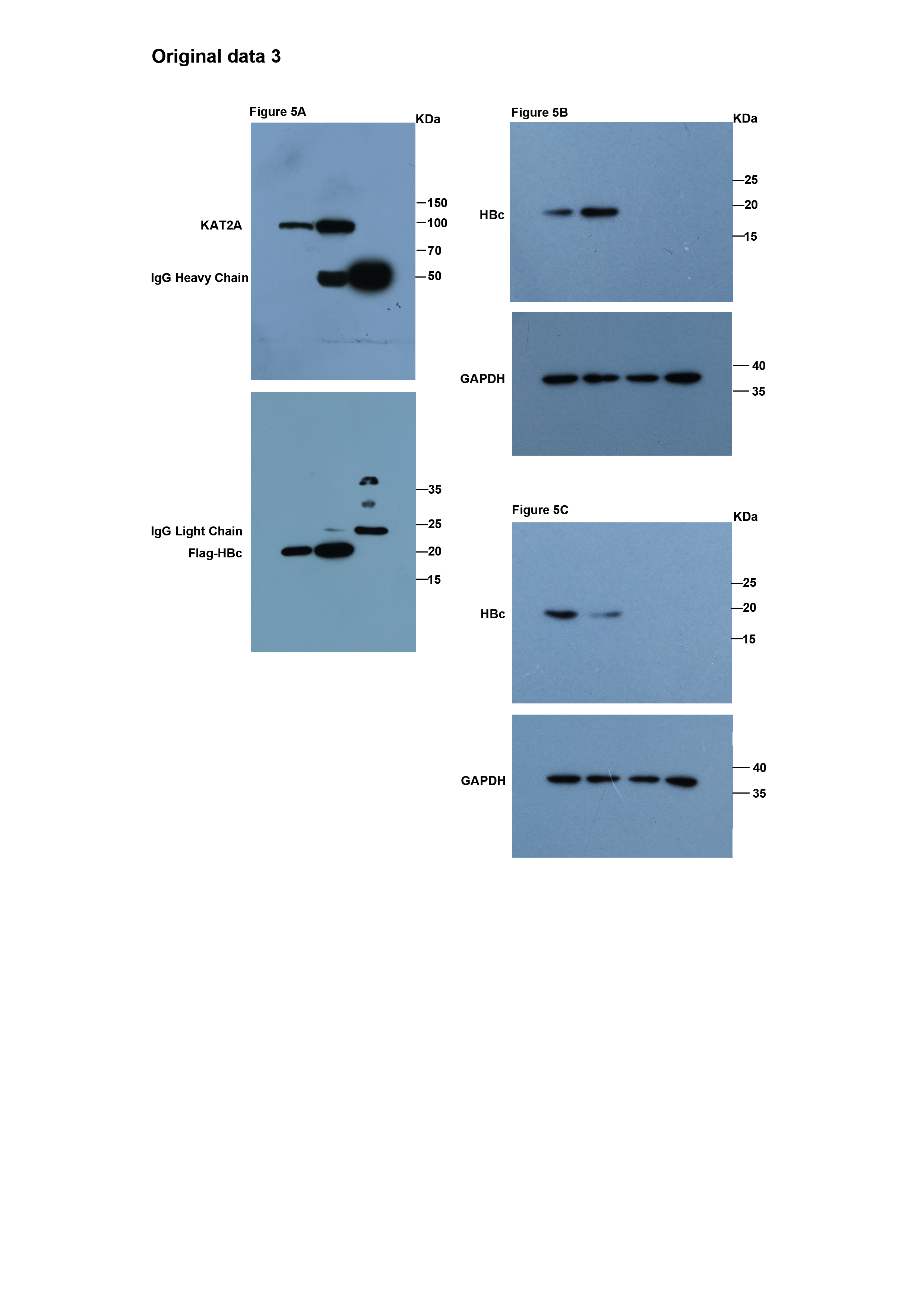

Supplement: Supplementary file 9 [file Image_8.TIF]

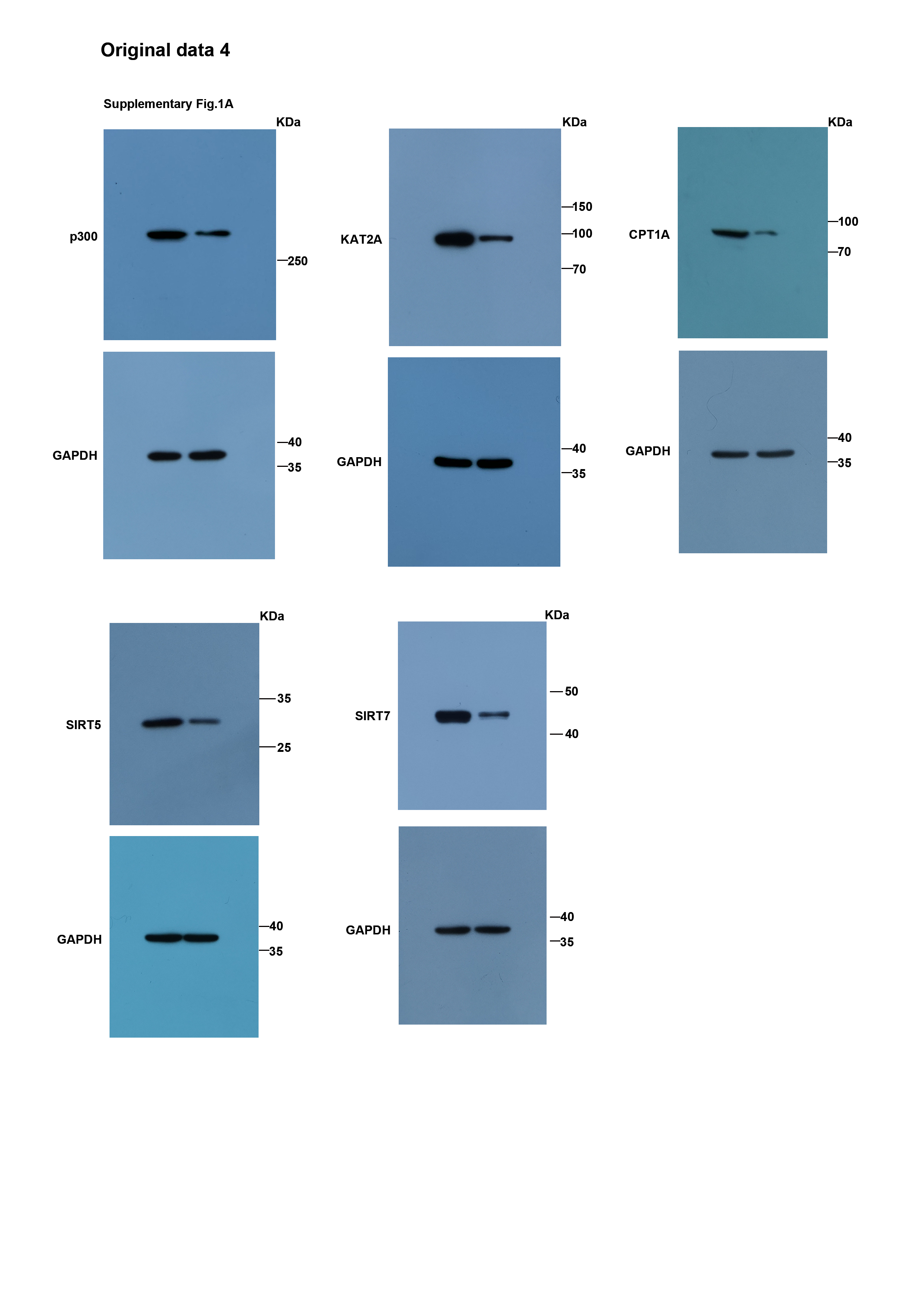

Supplement: Supplementary file 10 [file Image_9.TIF]

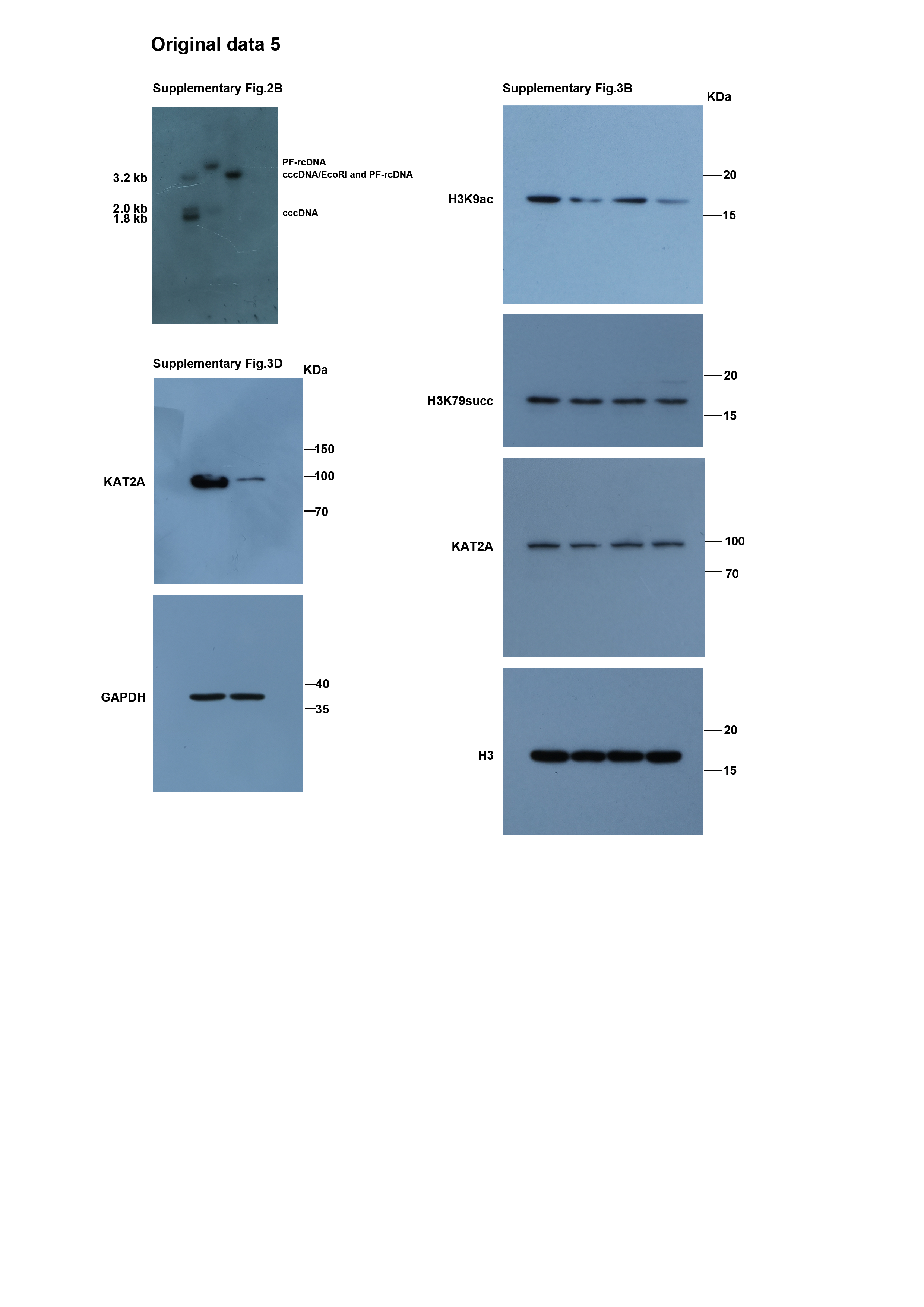

Supplement: Supplementary file 11 [file Image_10.TIF]

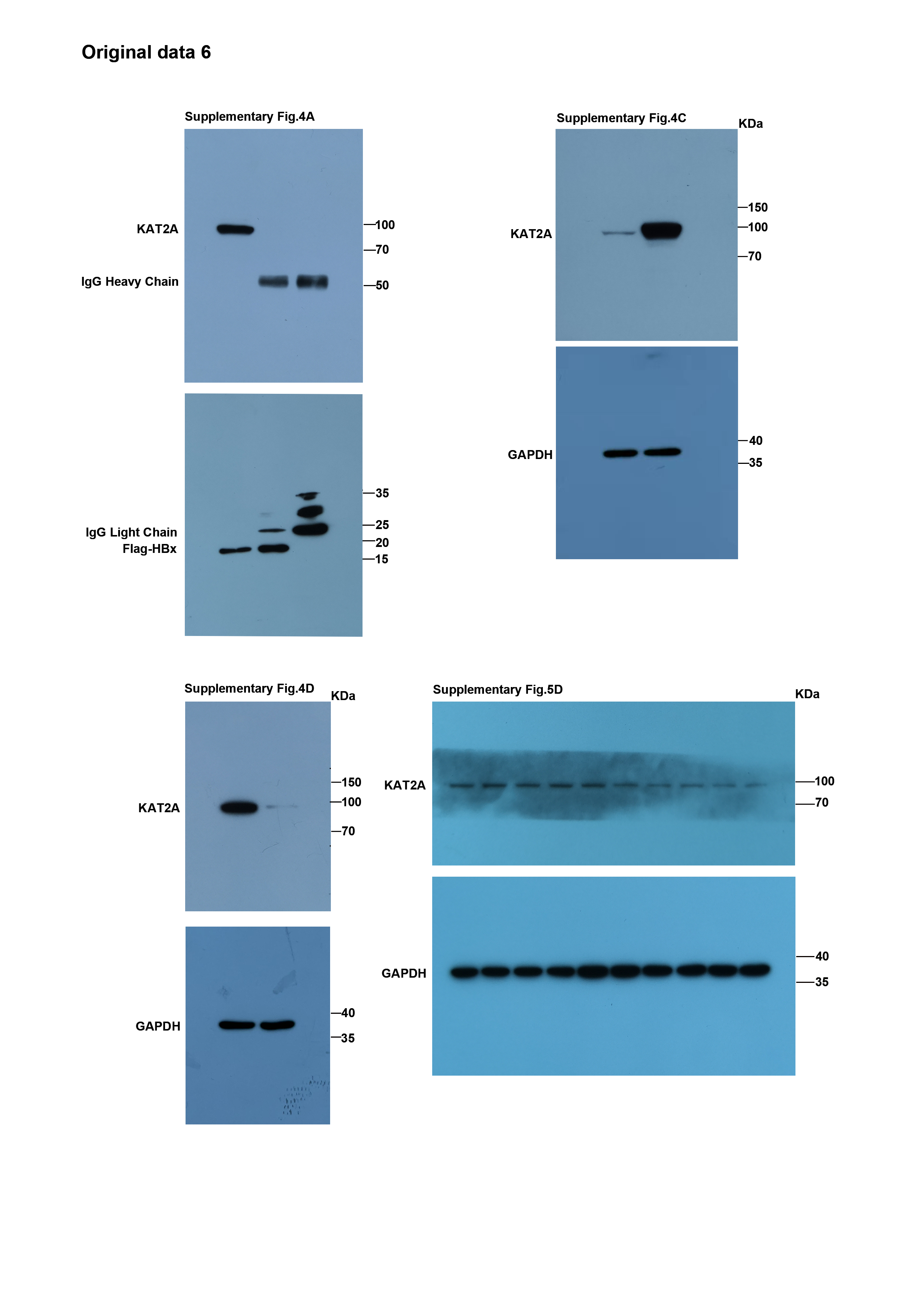

Supplement: Supplementary file 12 [file Image_11.TIF]
